# Assessment of photodynamic therapy with annatto and led for the treatment of halitosis in mouth-breathing children: Randomized controlled clinical trial

**DOI:** 10.1371/journal.pone.0307957

**Published:** 2024-09-03

**Authors:** Laura Hermida Bruno, Amanda Rafaelly Honório Mandetta, Ana Paula Taboada Sobral, Marcela Leticia Leal Gonçalves, Elaine Marcilio Santos, Ana Laura Fossati, Juliana Maria Altavista Sagretti Gallo, Pamella de Barros Motta, Alessandro Melo Deana, Anna Carolina Ratto Tempestini Horliana, Lara Jansiski Motta, Raquel Agnelli Mesquita Ferrari, Kristianne Porta Santos Fernandes, Sandra Kalil Bussadori

**Affiliations:** 1 Postgraduation Program in Biophotonic Medicine, Universidade Nove de Julho, UNINOVE, São Paulo, SP, Brazil; 2 Universidad Católica del Uruguay (UCU), Montevideo, Uruguay; 3 Postgraduation Program in Health and Environment, Universidade Metropolitana de Santos, Santos, Brazil; 4 School of Dentistry, Universidade Metropolitana de Santos, Santos, Brazil; 5 Postgraduation Program in Veterinary Medicine in the Coastal Environment, Universidade Metropolitana de Santos, Santos, Brazil; University of Catania: Universita degli Studi di Catania, ITALY

## Abstract

**Objective:**

To assess the effectiveness of antimicrobial photodynamic therapy (aPDT) employing an annatto-based (20%) dye combined with blue LED for the treatment of halitosis in mouth-breathing children.

**Materials and methods:**

Fifty-two children six to twelve years of age with diagnoses of mouth breathing and halitosis (score of ≥ 3 on portable breath meter) Breath Alert™ (Tanita Corporation®-Japan), were randomly allocated to two groups (n = 26). Group 1: brushing, dental floss and aPDT applied to middle third of the dorsum of the tongue. Group 2: brushing, dental floss and tongue scraper. Breath meter results before, immediately after treatment as well as seven and 30 days after treatment were compared. The hypothesis of normality in the data was discarded by the Shapiro-Wilk test (p < 0.05) and for statistical analysis the Wilcoxon and Mann-Whitney tests were used.

**Results:**

A significant difference was found between the pre-treatment reading and all other readings (p < 0.05) in both groups, suggesting the effectiveness of the proposed treatments. No significant difference was found between the post-treatment reading and two follow-up readings, suggesting the maintenance of the effect of treatment over time (p > 0.05). However, significant differences were found between groups for all post-treatment assessments (p < 0.0001 for all comparisons), indicating greater effectiveness with aPDT. No association was found between the initial reading and the presence of coated tongue.

**Conclusion:**

Antimicrobial photodynamic therapy using annatto and blue LED proved to be a viable therapeutic option for the treatment of halitosis in mouth-breathing children.

## Introduction

Nasal breathing is the main route for the entrance of air into the human organism and improves the quality of the air inhaled through filtering, heating and humidification, resulting in the protection of the airways. Mouth breathing is considered a pathological condition and can be caused by various factors, such as obstruction of the upper airways, flaccid facial muscles and harmful oral habits [1, 2]. Mouth breathing is generally a combination of nasal and oral breathing and the prevalence is reported to be higher than 50% of the population [3, 4]. Moreover, mouth breathing can lead to the evaporation of saliva, reducing its antibacterial effect and cleaning properties [5] and resulting in a considerable increase in halitosis [6–8].

Halitosis is a multifactorial condition with a variety of possible causes, although the decomposition of organic matter by proteolytic anaerobic bacteria is considered the main etiological factor. This metabolic process results in the production of volatile sulfur compounds, which are closely related to the characteristic odor associated with halitosis. This condition is classified in three categories: oral causes (accounting for 90% of cases), non-oral causes and other causes [9]. Oral halitosis is attributed to the microbial fermentation of food scraps, epithelial cells, saliva and blood accumulated on the dorsum of the tongue as well as in gingival and periodontal pockets [10].

The diagnosis of halitosis can be performed using direct measurements, such as the organoleptic test, portable sulfide monitors or gas chromatography, which is able to detect halitosis of any origin. Indirect methods can also be used, such as the BANA test, chemical sensors, the quantification of beta-galactosidase activity, salivary incubation test, ammonia monitoring, the ninhydrin method or polymerase chain reaction [7, 8, 10, 11].

Conventional methods for the control of halitosis are mainly based on the application of oral hygiene products containing bactericidal agents, the use of a tongue scraper, the treatment of caries and periodontal disease and the management of xerostomia [12]. Studies suggest that amine fluoride may also offer beneficial effects in the reduction of bad breath [8].

Antimicrobial photodynamic therapy (aPDT) has been employed for the control of halitosis [13–15]. This therapeutic modality has been widely investigated and administered in different health fields. Antimicrobial PDT consists of the administration of a photosensitizer combined with a light source at a specific wavelength and oxygen, leading to the generation of reactive oxygen species with the capacity to induce bacterial cell death [16–19].

The advantages of aPDT include the avoidance of bacterial resistance and the minimization of harm to the surrounding tissues. This is due to the fact that the antimicrobial effect is specifically directed at the areas treated with the photosensitizer and exposed to light, enabling rapid action in the biological target tissue, which varies with the intensity of the light energy applied. The likelihood of the development of bacterial resistance to aPDT is low due to the interaction of free radicals and singlet oxygen with a variety of bacterial cell structures and through different metabolic pathways, which hinders bacterial adaptation and increases the effectiveness of treatment [20].

For halitosis, the main etiological factor of which is anaerobic bacteria. aPDT has achieved promising results. The use of laser with a red wavelength combined with methylene blue dye as the photosensitizer has been effective at reducing hydrogen sulfide and the bacterial load on the dorsum of the tongue [13, 15, 18]. The use of annatto as a photosensitizer offers a significant advantage. This natural reddish dye can be employed with blue LEDs, which makes the protocol more accessible to dentists, most of whom already have this light source in their offices [21].

The advantages of these alternative approaches are the reduction in harm to tissues, the avoidance of bacterial resistance, effective, long-lasting halitosis treatment, the elimination of anaerobic bacteria related to this condition and the promotion of a better quality of life. Therefore, the aim of the present study was to assess the use of antimicrobial photodynamic therapy with annatto and blue LED for the reduction of halitosis in mouth-breathing children.

## Materials and methods

### Ethical aspects

This is a randomized, controlled clinical trial study followed the guidelines of Consolidated Standards of Reporting Trials (CONSORT) [22] and was approved by the Human Research Ethics Committee of Universidade Metropolitana de Santos-UNIMES (certificate number: 64510922.6.0000.5509). The study is available in Clinical Trials Registry (http://www.ClinicalTrials.gov) under number: NCT05590897.The study was conducted in accordance with the ethical precepts stipulated in the Declaration of Helsinki [23]. All information was contained in the statement of informed consent in accordance with Resolution 466/2012 e 510/2016 of the National Board of Health (Health Ministry, Federal District, Brazil). The guardians of the children agreed to participate by signing a statement of informed consent; two copies of which were signed: one for the guardian and one for the researchers. They were also informed that they could withdraw from the study at any time for any reason if they so wished. The researchers also had the ability to remove participants from the study if deemed necessary.

### Study design

The research protocol originally published 2023 [24], needed to undergo some modifications to ensure the feasibility of the study. One of these changes was the inclusion of children aged six to twelve, thus encompassing the population with mixed dentition. This age range was chosen based on previous prevalence studies that also used this age interval as a reference [25–27]. Initially, a microbiological assessment and a use of probiotics was planned in the project, but this step became unfeasible due to financial constraints. Given these limitations, the inclusion of two study groups focusing on the main outcome of the research was considered.

### Study population

The study included male and female mouth-breathing children who enter the dental clinic of Universidade Metropolitana de Santos (UNIMES), for treatment. The diagnosis of mouth breathing was performed using the calibrated Glatzel mirror test [28], water retention test [25] and a questionnaire [29]. The diagnosis of halitosis was performed using the portable Breath Alert™ (Tanita Corporation®-Japan), meter [29]. The participants were recruited from September 6, 2023, to November 15, 2023.

The Glatzel mirror is carried out by placing the mirror horizontally under the nose, lighted supported on the alae, and the participant was instructed to exhale. The halo of moisture on the mirror was traced with a marker. The measured area was interpreted as follows: < 30 mm = low nasal flow; 30–60 mm = medium nasal flow; > 60 m = high nasal flow. Participants with low nasal flow were classified as mouth breathers [25]. In the test of water retention, the participants were instructed to hold approximately 15 ml of water in the mouth for three minutes. Inability to hold the water for this period indicated the presence of mouth breathing [25].

The inclusion criteria were set as: female and male children six to twelve years of age with diagnoses of mouth breathing [25, 28, 29] and halitosis [29]. The exclusion criteria were nose breathers; individuals with dentofacial anomalies (e.g., hare lip and cleft palate); those in orthodontic and/or orthopedic treatment, those in oncological treatment; those with systemic (gastrointestinal, renal, or hepatic) conditions; pregnant girls, individuals who had undergone antibiotic treatment in the previous month, and individuals with fissured tongue.

### Sample size

The sample size was determined based on the primary outcome of our study. The initial sample size estimation was of 18 subjects per group for a significant level of 0.05 and an estimated test power of 95%. To account for the possible non-parametric distribution of the data, 15% more subjects must be added to each group [30]. Another 25% was added to account for possible dropouts, resulting in 26 subjects per group. G*Power 3.1.9.6 was used to perform the calculations.

### Randomization

Participants were allocated randomly into the two study groups using the simple randomization method. A computer-generated sequence (random.org; Randomness and Integrity Services, Dublin, Leinster, Ireland). Allocation concealment was ensured using sequentially numbered opaque sealed envelopes. Randomization and allocation were conducted by individuals not directly involved in the research, ensuring impartiality and minimizing potential influences of researchers on the participant assignment process to the study groups.

### Study groups

The participants were randomly allocated (block randomization) to two groups (n = 26 each). Group 1: toothbrushing, dental flossing and aPDT applied to the middle third of the dorsum of the tongue; Group 2: toothbrushing, dental flossing and the use of a tongue scraper. Comparisons of the breath meter results were performed before and immediately after treatment as well as seven and 30 days after treatment.

**Fig 1:**
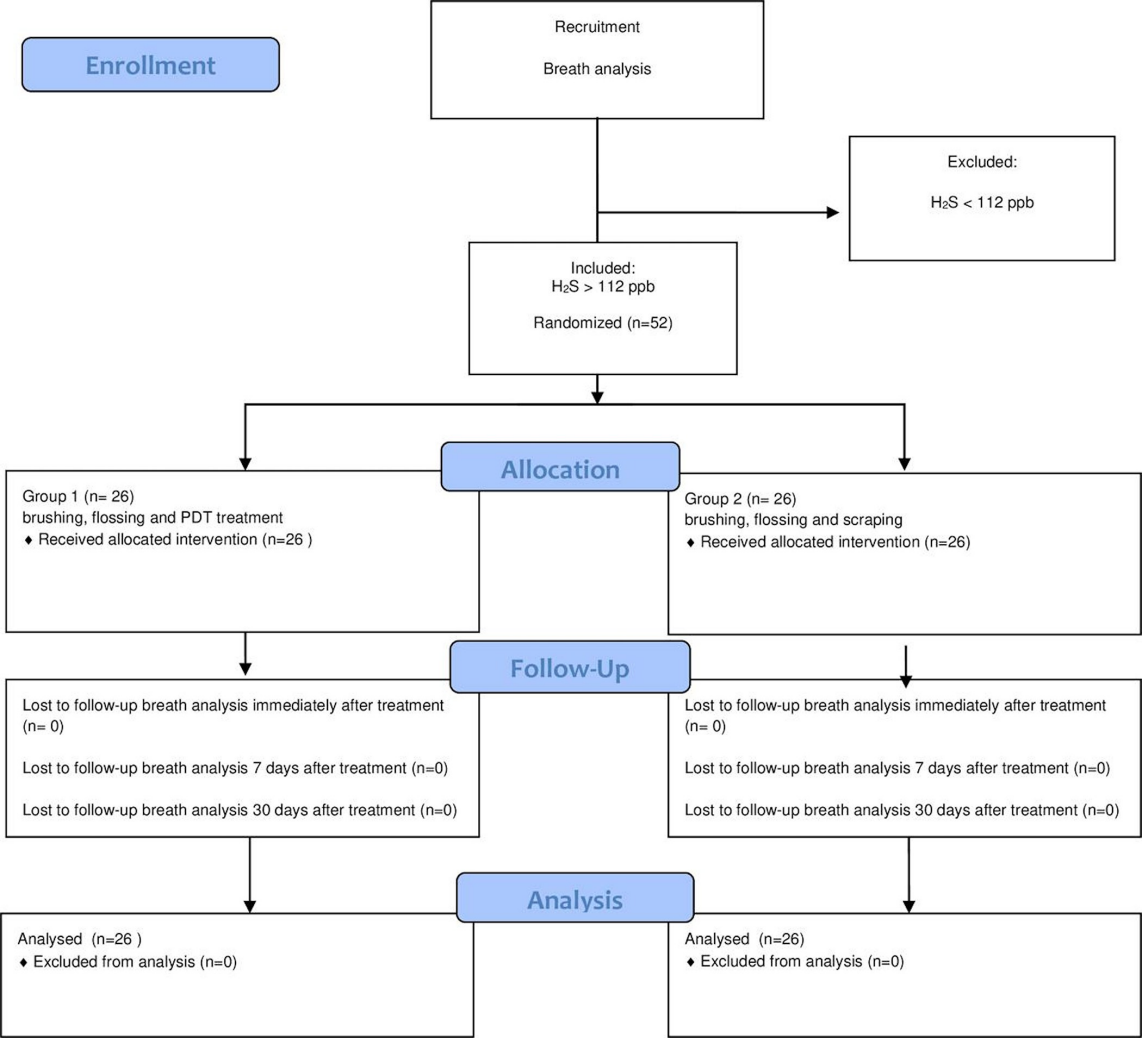
Study flowchart.

One hundred and twelve participants were evaluated, of which 52 were randomized, treated, and followed up (Fig 1).

### Interventions

#### Coated tongue assessment

The coated tongue index was calculated [29]. For such, the tongue was divided into nine regions. The presence of coated tongue in each region was assessed using a three-point scale: 0 = absence of coated tongue; 1 = presence of coated tongue with papillae visible; 2 = presence of coated tongue with papillae not visible. The scores attributed to each region were summed and divided by 18, representing the total number of regions analyzed. The result was than multiplied by 100 to generate the coated tongue index, which ranged from 0 to 100%.

#### Breath analysis

The Breath Alert™ (Tanita Corporation®-Japan) device was used following the manufacturer’s instructions and disinfected after each use. The device was shaken four or five times prior to each use to eliminate any residual odors. A “beep” was emitted upon opening the upper compartment of the device and a second “beep” was emitted when the participant blew into the frontal air input (air flow passage). After a third “beep”, breath odor was measured and scored on a scale of 0 to 8 points. If the letter ‘‘C” appeared, indicating an error, the procedure was repeated. A score ≥ 2 points was considered indicative of halitosis. The Breath Alert™ device measures the following volatile sulfur compounds:

- Hydrogen sulfide: Originating mainly from bacteria present on the dorsum of the tongue. Values higher than 112 ppb are indicative of halitosis.
- Methanethiol: Predominantly higher in periodontal pockets. Values up to 26 ppb are considered normal. Periodontal disease typically results in a high methanethiol/hydrogen sulfide ratio (>3:1).
- Dimethyl sulfide: The origin can be periodontal or systemic (intestinal, hepatic, pulmonary). This gas can also be temporarily caused by the ingestion of certain foods and beverages. An oral or systemic origin can be distinguished through the comparison of breath meter results with and without a cysteine challenge (16 mg of cysteine in 100 ml of distilled water). The perception threshold of dimethyl sulfide is low (8 ppb). Odors other than volatile sulfur compounds may appear at a peak prior to the theoretically first peak, which is that of hydrogen sulfide.

To ensure accurate readings, the participants were instructed to avoid the ingestion of garlic, onion and strong spices and not to use antiseptic mouthwash in the 48 hours prior to the assessment. On the day of the assessment, the participants could not eat anything in the two hours prior to the reading and were instructed to avoid coffee, mints, chewing gum, oral hygiene products and personal care products with fragrances (after shave, deodorant, perfume, creams and tonic). Brushing was to be performed only with water.

#### Antimicrobial photodynamic therapy (aPDT)

The Valo Cordless Ultradent^®^ LED photopolymerizing device was used, which is a dental office device with a radiometer operating in the 440 to 480 nm range, with irradiance of 450 mW/cm. During aPDT, only the participant and operator were present, both of whom used protective eyewear. The active LED tip was wrapped in disposable transparent plastic (PVC) film to avoid cross-contamination and for hygiene reasons. The operator wore proper apparel.

**Fig 2:**
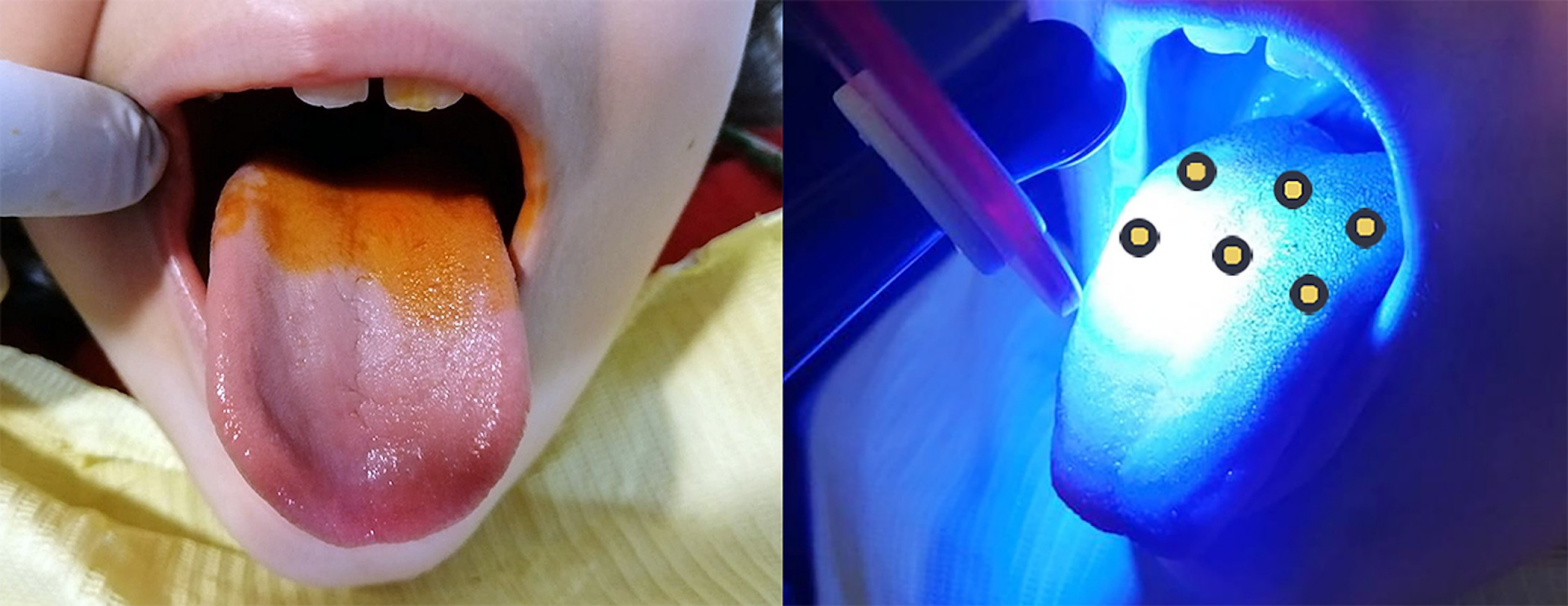
Antimicrobial PDT application points.

One session of aPDT was performed. The annatto photosensitizer was mixed at a concentration of 20% (Fórmula e Ação^®^, Brazil) in spray form, applied with a sufficient quantity to coat the middle third of the dorsum of the tongue (five sprays) and left for two minutes for incubation. The excess was removed with an aspirator. The surface of the tongue remained moist with the photosensitizer itself without the use of water. Six points were irradiated with a distance of 1 cm between points, considering the spread of the light and effectiveness of aPDT (Fig 2). The device was previously calibrated at a wavelength of 395–480 nm. Light was irradiated to ensure a beam area of 2 cm in diameter per point. Energy was 9.6 J and exposure time was 20 seconds per point. Table 1 displays the LED parameters.

**Table 1:** LED parameters.

Wavelength (nm)	395–480
**Operating mode**	Continuous
**Average radiant power (mW)**	480
**Polarization**	Random
**Opening diameter (cm)**	0.9
Irradiance at opening (mW/cm^2^)	762
**Beam profile**	Top hat
Irradiated area (cm^2^)	3.14
Target irradiance (mW/cm^2^)	153
**Exposure time (s)**	20
Fluency (J/cm^2^)	6.37
**Radiant energy (J)**	9.6
**Number of irradiated points**	6
Total irradiated area (cm^2^)	18.8
**Number of sessions**	1
**Total radiant energy (J)**	57.6

#### Tongue scraping

Tongue scraping was performed by the same operator for all participants. Posteroanterior movements were performed with the scraper on the dorsum of the tongue, followed by the cleaning of the scraper with gauze. The procedure was performed ten times on each participant to standardize mechanical removal.

#### Brushing with fluoridated toothpaste

All 52 participants were instructed through a lecture on how to perform toothbrushing with a fluoridated toothpaste (Colgate T®) and dental flossing three times per day after meals for 30 days. The Bass technique was taught, by which the bristles should be positioned at an approximate angle of 45° to the gingival pocket on both the free and proximal faces, with the performance of short, slightly circular vibrating movements [31, 32].

### Data analysis

Categorical data were analyzed using the chi-square test. The other datasets were examined for normality using the Shapiro-Wilk test. When nonparametric distribution was demonstrated, the analysis of multiple dependent groups was performed using the Friedman test, followed, when necessary, by the Wilcoxon test corrected by the Ryan-Holm Bonferroni stepwise correction. The Mann-Whitney test was used for independent datasets and also corrected by the Ryan-Holm Bonferroni stepwise procedure for multiple comparisons. Data with parametric distribution were submitted to analysis of variance (ANOVA), followed by the appropriate post-hoc test, when necessary. The significance level was set at α = 0.05.

## Results

### Age

The inferential analysis revealed no significant difference between the groups (p = 0.6620, Mann-Whitney test). Therefore, the two groups were matched in terms of age (Table 2).

**Table 2:** Descriptive analysis of age in study groups.

Groups	Median	Minimum	1^ST^ Quartile	4^TH^ Quartile	maximum
**1**	7.5	5	7	9	11
**2**	7.5	5	5	9	11

### Gender

The inferential analysis revealed no significant difference between the groups (p = 0.2669, chi-square test). Therefore, the two groups were matched in terms of gender (Table 3).

**Table 3:** Descriptive analysis of gender in study groups.

Groups/ Gender	F	M
1	10	16
2	15	11

**Fig 3:**
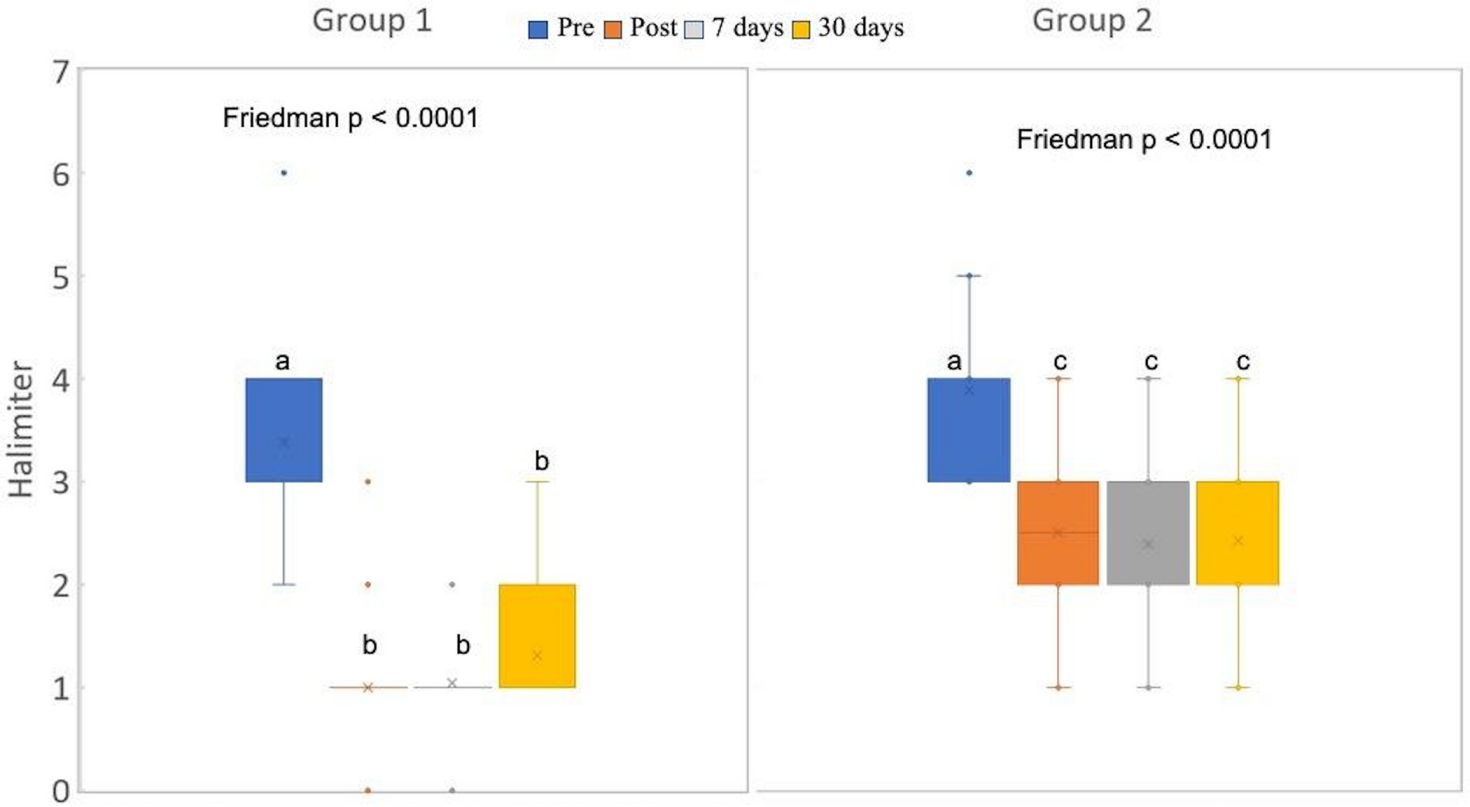
Boxplot of breath analyses. Different letters denote significant differences. A significant difference was found between the pre-treatment reading and all other readings (p < 0.05, Wilcoxson test) in both groups, suggesting the effectiveness of the proposed treatments. No significant difference was found between the post-treatment reading and two follow-up readings, suggesting the maintenance of the effect of treatment over time (p > 0.05, Wilcoxon test). No significant difference was found between groups at the initial reading, indicating good balance between the groups (p = 0.1316, Mann-Whitney test). However, significant differences were found between groups for all post-treatment assessments (p < 0.0001 for all comparisons, Mann-Whitney test), indicating greater effectiveness with aPDT.

The results of the comparison of the breath analysis between baseline and the readings performed immediately after treatment as well as seven and 30 days after treatment in Group 1 (aPDT) and Group 2 (tongue scraping) are displayed in the boxplot in Fig 3 The hypothesis of normality in the data was discarded by the Shapiro-Wilk test (p < 0.05).

**Fig 4:**
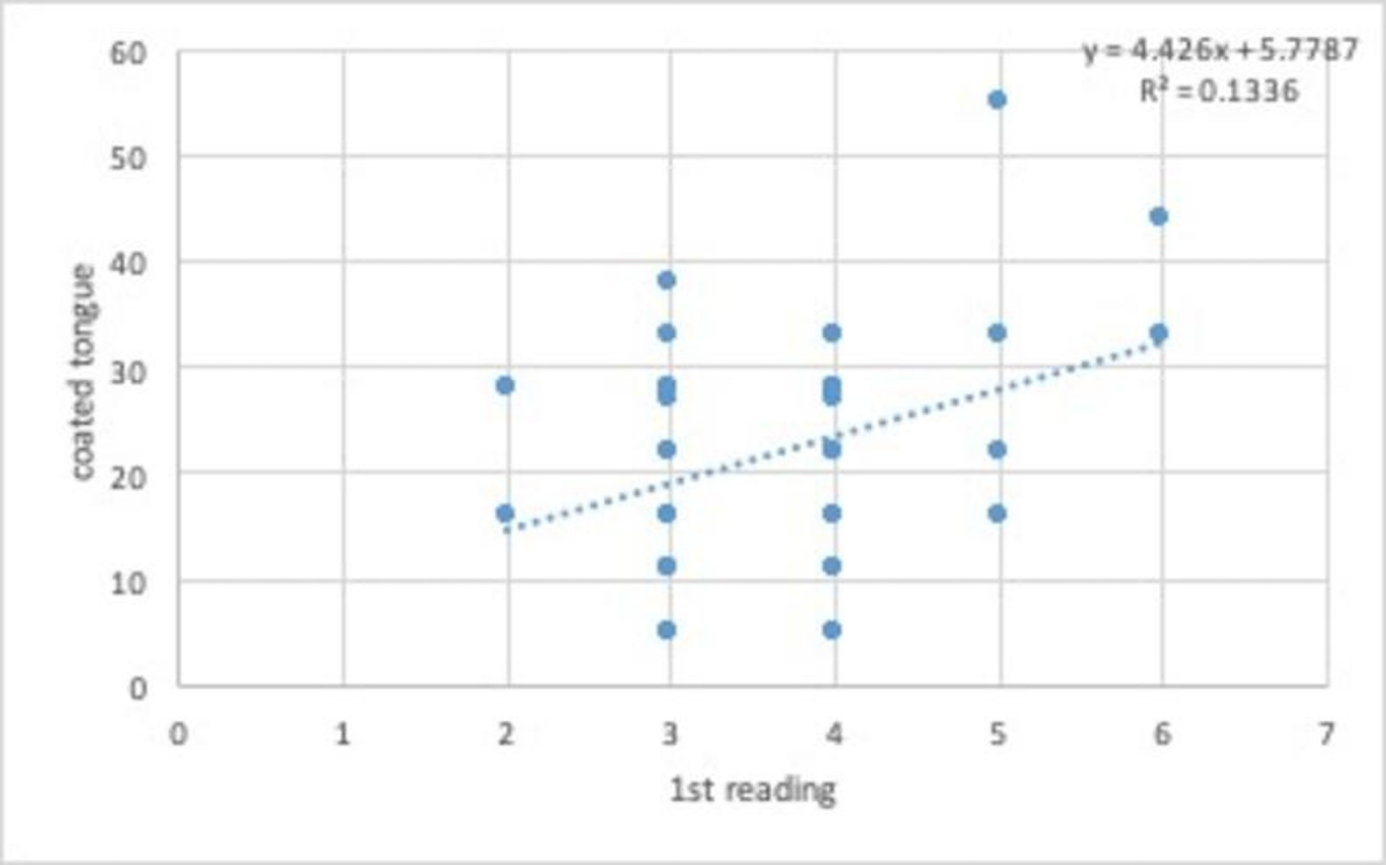
Dispersion graph of initial breath analysis and coated tongue.

No association was found between the initial reading and the presence of coated tongue. The dispersion graph (Fig 4) revealed a random pattern and the R^2^ value indicated a very weak correlation.

## Discussion

Halitosis is manifested as an unpleasant odor emanating from the oral cavity, nasal cavity and pharynx. Halitosis in children can exert an influence on psychological development in a critical period of social interaction [33]. Therefore, the treatment of halitosis is a subject that merits greater attention.

The main causes of halitosis of an intraoral origin in children are inadequate oral hygiene, a lack of daily dental flossing, changes in salivary flow, coated tongue and dental caries [26]. In a study investigating the dynamic change in the oral microbiota during the progression of halitosis in preschool children, the quantity of coated tongue and composition of the microbiome differed significantly between children with and without halitosis, even at the onset of clinical manifestations [33]. In the present study, however, no association was found between halitosis and the presence of coated tongue in mouth-breathing children. The most likely cause of halitosis in mouth-breathing individuals is the drying out of the surface of the mucosae due to the maintenance of the open mouth. Moreover, the evaporation of water from saliva may explain the association between mouth breathing and halitosis [34]. Despite the scarcity of evidence to sustain the association between mouth breathing and halitosis in children, some studies report such an association [34, 35].

The diagnosis of halitosis in children is crucial, as this condition can result in social problems and diminished quality of life and may also indicate more serious medical conditions [34]. In the present study, we employed the portable Breath Alert™ (Tanita Corporation®-Japan), device for the rapid, precise detection of halitosis in children. This device is a gas chromatograph with high diagnostic sensitivity and specificity that detects hydrogen sulfide and methanethiol, enabling the determination of the intensity and origin of halitosis [25].

The effects of aPDT with annato and blue LED in the reduction of halitosis have already been presented in other studies showing that the annatto extract contains several antioxidants and also generates the singlet oxygen, an oxidant molecule which plays an important role in microorganism killing during PDT. The aPDT is also a differentiated method in that it does not cause bacterial resistance or side effects, unlike conventional antimicrobial methods (e.g. mouthwash). It’s more comfortable than the tongue scraper which can cause discomfort [21].

According to a recent systematic review with meta-analysis, antimicrobial photodynamic therapy seems to be promising treatment for the control of halitosis [36]. A study that investigated the intervention in adolescents focused on administration to the dorsum of the tongue reported encouraging results, with a reduction in the concentration of volatile sulfur compounds [37]. Bad breath occurs when anaerobic bacteria in the oral cavity decompose cystine, cysteine and methionine, converting these amino acids into volatile sulfur compounds [38]. The present study suggests that aPDT can be considered a conservative, rapid, effective, noninvasive method for the treatment of halitosis in children and adolescents [37].

A recent systematic review found that, when administered alone, aPDT achieved better results that the use of a tongue scraper alone [32], which is in agreement with the findings of the present study. Both methods proved effective, as a significant reduction in halitosis was found after treatment in both groups. However, statistically significant differences were found between groups at all post-treatment assessments, suggesting greater effectivity with aPDT.

In the present study, statistically significant differences were found between the pre-treatment reading and all post-treatment readings, indicating a significant reduction in halitosis, which is compatible with results described in previous studies [15, 39]. However, it is noteworthy that the studies cited were conducted with adults and adolescents, whereas our study involved children with the mouth-breathing habit. Moreover, no significant differences were found between the reading made immediately after treatment and the two follow-up readings, suggesting the maintenance of the effect of treatment over time. This finding differs from results described in a study involving an adult population, in which the reduction in halitosis was not maintained after a period of seven days [39].

No significant differences between groups were found with regards to the gender or age of the participants. Few studies have investigated the association between halitosis and gender in children [36].

Although a recent Cochrane review reported evidence of low to very low quality with regards to the effectiveness of interventions for the management of halitosis, none of the studies in the review involved the use of aPDT [40]. This gap in the literature underscores the importance of further investigations to assess the potential of this therapeutic modality for the management of halitosis.

The results of the present study can contribute to clinical decision-making related to the use of aPDT with blue LED for the treatment of halitosis in daily clinical practice. This is particularly relevant, given that the majority of dentists already have this light source in their offices. Moreover, the use of annatto as a natural photosensitizer is an innovative approach. Due to the accessibility of both the light source and photosensitizer, this technique could be effectively and easily reproduced. When combined with adequate oral hygiene practices, such as toothbrushing, the use of a fluoridated toothpaste and dental flossing, aPDT may be effective for the reduction of halitosis in mouth-breathing children.

Considering the high incidence of mouth breathing in the young population and its oral repercussions, such as halitosis [34, 41], further studies are needed on the prevalence and causal factors of this condition as well as therapeutic options. Studies of this type have the potential to contribute to a significant improvement in the quality of life of this portion of the population.

Thinking about participants’ commitment to oral hygiene care at home, the researchers sent the oral hygiene instructions via WhatsApp file to all guardians, a face-to-face lecture was also held with the participants and their guardians, in which hygiene instructions were explained. Even in the face of these actions, we must be considered them as a limitation of the study the fact that participants were not professionally monitored at home.

## Conclusion

Antimicrobial photodynamic therapy using an annatto-based (20%) dye and blue LED proved to be a viable therapeutic option for the treatment of halitosis in mouth-breathing children, with the maintenance of the effect of treatment over time. Halitosis does not seem to be associated with the presence of coated tongue in mouth-breathing children.

## Supporting information

S1 Checklist CONSORT 2010 checklist of information to include when reporting a randomised trial*. (DOCX)

S1 File CONSORT checklist. (DOC)

S2 File Feedback from the Ethics Committee in original language. (PDF)

S3 File Feedback from the Ethics Committee in English. (PDF)

S4 File Study protocol in original language. (PDF)

S5 File Study protocol in English. (PDF)

S6 File Statement consent in original language. (PDF)

S7 File Statement consent in English. (PDF)

S8 File ClinicalTrials. (PDF)

S1 Data (XLSX)

S2 Data (XLSX)
