# Supplementary material for: Assessment of photodynamic therapy with annatto and led for the treatment of halitosis in mouth-breathing children: Randomized controlled clinical trial
Source: PLoS One. 2024 Sep 3;19(9):e0307957. doi: 10.1371/journal.pone.0307957 (PMC11371243; doi:10.1371/journal.pone.0307957)
Supplement: S2 File — (PDF) [file pone.0307957.s003.pdf]

PARECER CONSUBSTANCIADO DO CEP

DADOS DO PROJETO DE PESQUISA

Título da Pesquisa: ESTUDO COMPARATIVO ENTRE A TERAPIA FOTODINÂMICA E USO DE PROBIÓTICOS NA REDUÇÃO DA HALITOSE EM CRIANÇAS RESPIRADORAS ORAIS: ENSAIO CLÍNICO CONTROLADO E RANDOMIZADO

Pesquisador: ANA PAULA TABOADA SOBRAL

Área Temática:

Versão: 1

CAAE: 64510922.6.0000.5509

Instituição Proponente: CENTRO DE ESTUDOS UNIFICADOS BANDEIRANTE

Patrocinador Principal: Financiamento Próprio

DADOS DO PARECER

Número do Parecer: 5.728.471

Apresentação do Projeto:

"Halitose é um termo que define qualquer odor ou mau-cheiro proveniente da cavidade oral, que pode apresentar origem local ou sistêmica [1]. O mau odor oral pode ser atribuído a uma variedade de produtos oriundos do metabolismo de aminoácidos bacterianos. A halitose pode ser classificada como genuína, pseudo-halitose e halitofobia. A halitose genuína se divide em halitose fisiológica (causada principalmente pela saburra ligual) e halitose patológica que pode ser oral (doenças bucais) ou extra-oral (doenças sistêmicas). A pseudo-halitose consiste na autopercepção do paciente que relata a presença de mau hálito mesmo quando ele não é percebido por outras pessoas e não é diagnosticado clinicamente. Já a halitofobia é uma condição em que, mesmo sem evidências clínicas ou sociais, e após os tratamentos específicos, o paciente se queixa de mau hálito.A prevalência da halitose é alta, sendo possível encontrar na literatura valores acima de 50% e é considerada um importante fator social, pois interfere nas relações interpessoais. Além de gerar preocupações relacionadas à saúde física do indivíduo, pode provocar alterações psicológicas, conduzindo a uma barreira social. Nesse contexto de importância social e biológica a prevalência e associações de halitose em populações pediátricas tem sido investigada no mundo com estimativas variadas. Estudos recentes voltam um olhar atento às crianças respiradoras orais e demonstram que esse grupo apresenta aumento significativo do nível de halitose em comparação aos respiradores nasais. A halitose classificada como oral se origina na boca ou nas vias aéreas

Endereço: Av Conselheiro Nébias 536
Bairro: Encruzilhada **CEP:** 11.045-002
UF: SP **Município:** SANTOS
Telefone: (13)3228-3400 **Fax:** (13)3226-3400 **E-mail:** fernanda.agnelli@unimes.br

superiores e resulta da decomposição da matéria orgânica que origina- se de lascas de células epiteliais retidas no porção posterior do dorso da língua que ocorre, entre outros fatores, pela redução do fluxo salivar e/ou desequilíbrio hídrico e pelo ataque microbiano no meio bucal que favorecem o crescimento de bactérias proteolíticas e, consequentemente, resultam na produção de compostos voláteis de enxofre relacionados ao odor característico. Quando a taxa de fluxo salivar diminui, a contagem bacteriana e a halitose na cavidade oral aumentam. A mudança da respiração nasal para respiração oral, causa adaptação, alterações nas arcadas dentárias e tecidos circundantes, como alterações anatômicas do palato e ressecamento da superfície da mucosa. A secagem superficial da mucosa em crianças respiradoras, que é uma das queixas principais em indivíduos que respiram pela boca, pode estar relacionada à halitose. Pacientes com respiração oral por hipertrofia adenotonsilar avaliadosapresentaram maiores taxas de halitose quando comparado aos grupos de tratamento (cirurgia) e controle (respiradores nasais).Os Compostos Sulfurosos Voláteis (CSV) são componentes químicos que estão relacionados com a presença de halitose; o sulfidreto (relacionado à saburra lingual), o metilmercaptano (relacionado à bolsas periodontais) e o dimetilsulfeto (relacionado à alterações sistêmicas). Existem diferentes métodos de diagnóstico de halitose: a avaliação clínica, conhecida como teste organoléptico, um método subjetivo que consiste em sentir o cheiro exalado pela boca e pelo nariz, e em seguida, quantificar esse odor com o uso de uma escala. Os CVS podem ser medidos com o uso de monitores de sulfeto e cromatografia gasosa. O dispositivo portátil Breath-Alert (BA) tem sido cada vez mais empregado na prática clínica para o diagnóstico da halitose devido à sua facilidade de uso e baixo custo. Em crianças, que necessitam de exames rápidos e práticos, o BA é uma ferramenta para a detecção de halitose na prática da odontopediatria que demonstra alta sensibilidade e especificidade. Os tratamentos convencionais utilizados no controle da halitose consistem basicamente no uso de dentifrícios e colutórios contendo substâncias bactericidas, uso de raspador lingual, tratamento das lesões de cárie e da doença periodontal, além do controle da xerostomia. Alguns estudos sugerem que o fluoreto de amina tem efeito positivo na diminuição da halitose. Estudos mostram que tratamentos alternativos, como a Terapia Fotodinâmica Antimicrobiana (aPDT) e probióticos, tem sido empregado na tentativa de controlar a halitose. A aPDT é um tratamento no qual é utilizado um agente fotossensibilizador, um corante, que na presença de luz, produz radicais livres de oxigênio levando à morte celular, no caso da halitose que tem o principal fator etiológico relacionado com a presença de bactérias anaeróbias, essa terapia apresentou resultados positivos (com uso de laser vermelho e azul de metileno) na redução de sulfeto de hidrogênio, bem como na redução da carga bacteriana no dorso da língua. O corante

Urucum vem sendo avaliado como um fotossensibilizador em estudos relacionados a halitose. Extraído da semente da Bixa orellana, uma planta nativa do Brasil, urucum é aceito pela Organização Mundial da Saúde (OMS), devido ao fato de não ser tóxico. Possui importante atividade antioxidante e antimicrobiana, e estudos recentes têm demonstrado seu potencial como agente terapêutico e corante natural. Os probióticos são definidos como microrganismos que proporcionam efeitos benéficos para a saúde do hospedeiro quando absorvidos pelo mesmo. São frequentemente utilizados em alimentos e produtos fermentados, além de serem utilizados em manipulações farmacêuticas. As pesquisas mostram resultados positivos no uso de probióticos no controle da halitose e sugerem que eles possam favorecer a eliminação de alguns microrganismos indesejáveis e promover a recolonização da microbiota do indivíduo.As vantagens de abordagens alternativas como aPDT e urucum e administração de probióticos para a redução ou eliminação da halitose na odontopediatria são técnicas menos invasivas que lançam uso de componentes naturais como urucum que pode reduzir os danos aos tecidos orais e evitar resistência bacteriana. É desafiador desenvolver, pesquisar estabelecer um protocolo de tratamento para halitose que possa ser eficaz, não traumático nessa faixa etária e duradouro, eliminando as bactérias anaeróbias relacionadas à essa condição e possivelmente pelo equilíbrio sistêmico, restabelecer a microbiota do dorso da língua, a fim de promover uma melhora na qualidade
de vida integral do indivíduo. O uso de probióticos na odontologia apresenta um tratamento inovador, capaz de modificar a microbiota oral, como uma alternativa ao uso de antibióticos e outros produtos antimicrobianos.O tratamento da halitose é um tema que ainda precisa de atenção e os resultados deste estudo podem apoiar a tomada de decisões dos profissionais de saúde em relação ao uso de probióticos e à aPDT usando LEDs azuis para tratar a halitose em seu cotidiano, já que a maioria dos dentistas já possui essa fonte de luz em seus consultórios e o medidor portátil é de baixo custo para aquisição. Além disso, a utilização do extrato urucum como fotossensibilizador é inovador. Uma vez que se trata de uma fonte de luz e um fotossensibilizador acessível, espera-se que este tratamento seja reproduzido clinicamente com eficácia e facilidade. Espera-se que o
uso de probióticos e o uso da aPDT sejam eficazes na diminuição da halitose em crianças respiradoras orais." As informações elencadas nos campos “Apresentação do Projeto” foram retiradas do Arquivo Informações básicas de Pesquisa (PB_INFORMAÇOES_BÁSICAS_DO_PROJETO_2036317.pdf) de 19/10/2022, versão 1.

Objetivo da Pesquisa:

"O objetivo do presente estudo é verificar se o tratamento com aPDT, utilizando extrato de urucum

como fotossensibilizador e o LED azul como fonte de luz, é eficaz na redução em crianças respiradoras oral". As informações elencadas nos campos “Objetivo da Pesquisa” foram retiradas do Arquivo Informações básicas de Pesquisa (PB_INFORMAÇOES_BÁSICAS_DO_PROJETO_2036317.pdf) de 19/10/2022, versão 1.

Avaliação dos Riscos e Benefícios:

Riscos: "O paciente poderá apresentar desconforto durante a escovação dental".

Benefícios: "Os voluntários e seus responsáveis participarão das atividades de educação em saúde bucal com aconselhamento de alimentação e higiene. Os voluntários terão a boca examinada e se houver necessidade serão encaminhadas para tratamento odontológico"

As informações elencadas nos campos “Avaliação dos Riscos e Benefícios” foram retiradas do Arquivo Informações básicas de Pesquisa (PB_INFORMAÇOES_BÁSICAS_DO_PROJETO_2036317.pdf) de 19/10/2022, versão 1.

Comentários e Considerações sobre a Pesquisa:

Trata-se de um ensaio clínico controlado e randomizado.

Considerações sobre os Termos de apresentação obrigatória:

Os termos estão de acordo com as Resoluções do CNS 466/12 e 510/16.

Conclusões ou Pendências e Lista de Inadequações:

Projeto aprovado.

Considerações Finais a critério do CEP:

Este parecer foi elaborado baseado nos documentos abaixo relacionados:

Tipo Documento	Arquivo	Postagem	Autor	Situação
Informações Básicas do Projeto	PB_INFORMAÇÕES_BÁSICAS_DO_PROJETO_2036317.pdf	19/10/2022 19:35:23		Aceito
TCLE / Termos de Assentimento / Justificativa de Ausência	TCLEProjetoHalitose.docx	19/10/2022 19:34:20	ANA PAULA TABOADA SOBRAL	Aceito
Projeto Detalhado	ProjetoHalitoseUNIMES.pdf	19/10/2022	ANA PAULA	Aceito

/ Brochura Investigador	ProjetoHalitoseUNIMES.pdf	19:30:21	TABOADA SOBRAL	Aceito
Folha de Rosto	folhaDeRostohalitose.pdf	19/10/2022 19:26:26	ANA PAULA TABOADA SOBRAL	Aceito

Situação do Parecer:
Aprovado

Necessita Apreciação da CONEP:
Não

SANTOS, 27 de Outubro de 2022

Assinado por:
Marcela Leticia Leal Gonçalves
(Coordenador(a))
