# Supplementary material for: Assessment of photodynamic therapy with annatto and led for the treatment of halitosis in mouth-breathing children: Randomized controlled clinical trial
Source: PLoS One. 2024 Sep 3;19(9):e0307957. doi: 10.1371/journal.pone.0307957 (PMC11371243; doi:10.1371/journal.pone.0307957)
Supplement: S3 File — (PDF) [file pone.0307957.s004.pdf]

Machine Translated by Google

UNIVERSITY SANTOS METROPOLITAN - UNIMES

CONSUBSTANTIATED OPINION OF THE CEP

RESEARCH PROJECT DATA

Search Title: COMPARATIVE STUDY BETWEEN PHOTODYNAMIC THERAPY AND THE USE OF PROBIOTICS FOR REDUCING HALITOSIS IN RESPIRATORY CHILDREN ORAL: CONTROLLED AND RANDOMIZED CLINICAL TRIAL

Researcher: ANA PAULA TABOADA SOBRAL

Thematic Area:

Version: 1

CAAE: 64510922.6.0000.5509

Proposing Institution: BANDEIRANTE UNIFIED STUDIES CENTER

Main Sponsor: Own Financing

OPINION DATA

Opinion Number: 5,728,471

Project Presentation:

"Halitosis is a term that defines any odor or bad smell originating from the oral cavity, which may have local or systemic origin [1]. Bad oral odor can be attributed to a variety of products arising from amino acid metabolism bacterial. Halitosis can be classified as genuine, pseudo-halitosis and halitophobia. Genuine halitosis is divided into physiological halitosis (caused mainly by tongue coating) and pathological halitosis which can be oral (oral diseases) or extra-oral (systemic diseases) . Pseudo-halitosis consists of the self-perception of the patient who reports the presence of bad breath even when it is not noticed by other people and is not clinically diagnosed. Halitophobia is a condition in which, even without clinical or social evidence, and after specific treatments, the

patient complains of bad breath. The prevalence of halitosis is high, values above 50% can be found in the literature and it is considered an important social factor, as it interferes with interpersonal relationships.

In addition to generating concerns related to the individual's physical health, it can cause psychological changes, leading to a social barrier. In this context of social and biological importance, the prevalence and associations of halitosis in pediatric populations have been investigated worldwide with

varying estimates. Recent studies take a closer look at mouth-breathing children and demonstrate that this group presents a significant increase in the level of halitosis compared to nasal breathers. Halitosis classified as oral originates in the mouth or airways

Address: Av Conselheiro Nébias 536
Neighborhood: Crossroads **ZIP CODE:** 11.045-002
UF: SP **Municipality:** SANTOS
Telephone: (13)3228-3400 **Fax:** (13)3226-3400 **Email:** fernanda.agnelli@unimes.br

Machine Translated by Google

superior areas and results from the decomposition of organic matter that originates from slivers of epithelial cells retained in the posterior portion of the back of the tongue that occurs, among other factors, due to reduced salivary flow and/or water imbalance and microbial attack in the oral environment that favor the growth of proteolytic bacteria and, consequently, result in the production of volatile sulfur compounds related to the characteristic odor. When the salivary flow rate decreases, the bacterial count and halitosis in the cavity

oral increase. The change from nasal breathing to oral breathing causes adaptation, changes in the dental arches and surrounding tissues, such as anatomical changes in the palate and dryness of the mucosal surface. Superficial drying of the mucosa in breathing children, which is one of the main complaints in individuals who breathe through the mouth, may be related to halitosis. Patients with oral breathing due to adenotonsillar hypertrophy evaluated presented higher rates of halitosis when compared to the treatment (surgery) and control (nasal breathers) groups. Volatile Sulfur Compounds (CSV) are chemical components that are related to the presence of halitosis; sulfide (related to tongue coating), methylmercaptan (related to periodontal pockets) and dimethylsulfide (related to systemic changes). There are different methods of diagnosing halitosis: clinical assessment, known as organoleptic test, a subjective method that consists of feeling the smell exhaled through the mouth and nose, and then quantifying this odor using a scale. CVS can be measured using sulfide monitors and gas chromatography. The Breath-Alert (BA) portable device has been increasingly used in clinical practice for diagnosing halitosis due to its ease of use and low cost. In children, who require quick and practical exams, BA is a tool for detecting halitosis in pediatric dentistry practice that demonstrates high sensitivity and specificity.

Conventional treatments used to control halitosis basically consist of the use of

toothpastes and mouthwashes containing bactericidal substances, use of tongue scrapers, treatment of caries lesions and periodontal disease, in addition to controlling xerostomia. Some studies suggest that amine fluoride has a positive effect on reducing halitosis. Studies show that alternative treatments, such as Antimicrobial Photodynamic Therapy (aPDT) and probiotics, have been used in an attempt to control halitosis. aPDT is a treatment in which a photosensitizing agent is used, a dye, which in the presence of light, produces free oxygen radicals leading to cell death, in the case of halitosis, which has the main etiological factor related to the presence of anaerobic bacteria, This therapy showed positive results (using red and methylene blue lasers) in reducing hydrogen sulfide, as well as reducing the bacterial load on the back of the tongue. The dye

Machine Translated by Google

Annatto has been evaluated as a photosensitizer in studies related to halitosis. Extracted from

Bixa orellana seed, a plant native to Brazil, annatto is accepted by the World Health Organization (WHO), due to the fact that it is non-toxic. It has important antioxidant and antimicrobial activity, and recent studies have demonstrated its potential as a therapeutic agent and natural dye. Probiotics are defined as microorganisms that provide beneficial effects on the health of the host when absorbed by the host. They are frequently used in foods and fermented products, in addition to being used in pharmaceutical manipulations. Research shows positive results in the use of probiotics to control halitosis and suggests that they can favor the elimination of some undesirable microorganisms and promote the recolonization of the individual's microbiota. The advantages of alternative approaches such as aPDT and annatto and administration of probiotics for reduction or elimination of halitosis in pediatric dentistry are less invasive techniques that use natural components such as annatto that can reduce damage to oral tissues and prevent bacterial resistance. It is challenging to develop, research and establish a treatment protocol for halitosis that can be effective, non-traumatic in this age group and long-lasting, eliminating anaerobic bacteria related to this condition and possibly through systemic balance, reestablishing the microbiota on the back of the tongue, in order to promote an improvement in the individual's overall quality of life. The use of probiotics in dentistry presents an innovative treatment, capable of modifying the oral microbiota, as an alternative to the use of antibiotics and other antimicrobial products. The treatment of halitosis is a topic that still needs attention and the results of this study can support the healthcare professionals' decision-making regarding the use of probiotics and aPDT using blue LEDs to treat halitosis in their daily lives, as most dentists already have this light source in their offices and the portable meter is low cost for acquisition. Furthermore, the use of annatto extract as a photosensitizer is innovative. As

it is an affordable light source and photosensitizer, this treatment is expected to be clinically replicated effectively and easily. It is expected that the use of probiotics and the use of aPDT will be effective in reducing halitosis in mouth-breathing children." The information listed in the "Project Presentation" fields were taken from the Basic Research Information File (PB_INFORMAÇOES_BÁSICAS_DO_PROJETO_2036317.pdf) of 19 / 10/2022, version 1.

Research Objective:

"The objective of the present study is to verify whether treatment with aPDT, using annatto extract

Machine Translated by Google

as a photosensitizer and the blue LED as a light source, it is effective in reducing mouth-breathing children." , version

1.

Assessment of Risks and Benefits: Risks:

"The patient may experience discomfort during tooth brushing."

Benefits: "Volunteers and their guardians will participate in oral health education activities with nutrition and hygiene advice. Volunteers will have their mouths examined and, if necessary, will be referred for dental treatment"

The information listed in the fields "Assessment of Risks and Benefits" was taken from the Basic Research Information File (PB_INFORMAÇOES_BÁSICAS_DO_PROJETO_2036317.pdf) of 10/19/2022, version 1.

Comments and Considerations about the Research:
This is a controlled and randomized clinical trial.

Considerations about the Mandatory Presentation Terms: The terms are in accordance with CNS Resolutions 466/12 and 510/16.

Conclusions or pending issues and list of inadequacies:

Project approved.

Final Considerations at the discretion of the CEP:

This opinion was prepared based on the documents listed below:

Document Type File PB_INFORMAÇÕES_BÁSICAS_DO_P Basic		Post	Author	Situation
Terms of TCLEProjetoHalitose.docx Assent / Justification of Absence	Information of the Project ROJETO_2036317.pdf TCLE / Detailed Project	10/19/2022 19:35:23		Accepted
ProjetoHalitoseUNIMES.pdf		10/19/2022 19:34:20	ANA PAULA TABOADA SOBRAL	Accepted
		10/19/2022	ANA PAULA	Accepted

Machine Translated by Google

/ Brochure Investigator	ProjetoHalitoseUNIMES.pdf	19:30:21	TABOADA SOBRAL	Accepted
Title Page	sheetOfRostohalitosis.pdf	10/19/2022 19:26:26	ANA PAULA TABOADA SOBRAL	Accepted

Status of the Opinion:

Approved

Requires CONEP Appraisal:

No

SANTOS, October 27, 2022

Signed by:

Marcela Leticia Leal Gonçalves
(Coordinator)

Address: Av Conselheiro Nébias 536
Neighborhood: Crossroads **ZIP CODE:** 11.045-002
UF: SP **Municipality:** SANTOS
Telephone: (13)3228-3400 **Fax:** (13)3226-3400 **Email:** fernanda.agnelli@unimes.br
