# Supplementary material for: Assessment of photodynamic therapy with annatto and led for the treatment of halitosis in mouth-breathing children: Randomized controlled clinical trial
Source: PLoS One. 2024 Sep 3;19(9):e0307957. doi: 10.1371/journal.pone.0307957 (PMC11371243; doi:10.1371/journal.pone.0307957)
Supplement: S4 File — (PDF) [file pone.0307957.s005.pdf]

PROJETO DE PESQUISA

ESTUDO COMPARATIVO ENTRE A TERAPIA FOTODINÂMICA E USO DE PROBIÓTICOS NA REDUÇÃO DA HALITOSE EM CRIANÇAS RESPIRADORAS ORAIS: ENSAIO CLÍNICO CONTROLADO E RANDOMIZADO

Pesquisadora Responsável: Profa. Dra. Ana Paula Taboada Sobral

Santos

2022

RESUMO

Halitose é um termo que define qualquer odor ou mau-cheiro proveniente da cavidade oral, que pode apresentar origem local ou sistêmica. Umas das causas da halitose local ou oral é a redução do fluxo salivar e ressecamento bucal, tida também como uma das queixas mais comuns entre os respiradores orais. Este projeto tem como objetivo verificar a eficácia da terapia fotodinâmica antimicrobiana (aPDT) e uso de probióticos no tratamento da halitose em crianças respiradores orais. Para o estudo serão selecionadas 52 crianças com idade entre 7 e 12 anos que após entrevista e exame clínico inicial tenham diagnóstico de respiradores orais e halitose. Os participantes serão divididos em 04 grupos. Grupo 1: tratamento com escovação, fio dental e raspador de língua; Grupo 2: escovação, fio dental e aPDT aplicada na região de dorso e terço médio da língua; Grupo 3: escovação fio dental e probióticos; Grupo 4: escovação, fio dental, aPDT e probióticos. Em todos os grupos a halimetria e análise microbiológica da saburra lingual serão realizadas antes, imediatamente após o tratamento e sete dias após o tratamento. A análise quantitativa será realizada por contagem de bactérias por unidades formadoras de colônias por mililitro e PCR em tempo real. A normalidade dos dados será aferida através do teste Shapiro-Wilk, e, no caso de normalidade será aplicado o teste de Análise de Variância (ANOVA), e, no caso de dados não-paramétricos, será utilizado o teste Kruskal-Wallis. Para analisar os resultados de cada tratamento nos dois períodos do estudo será utilizado o teste de Wilcoxon.

Descritores: Halitose, Terapia Fotodinâmica, *Bixa orellana*, Probióticos

1. Introdução

Halitose é um termo que define qualquer odor ou mau-cheiro proveniente da cavidade oral, que pode apresentar origem local ou sistêmica [1]. O mau odor oral pode ser atribuído a uma variedade de produtos oriundos do metabolismo de aminoácidos bacterianos. A halitose pode ser classificada como genuína, pseudo-halitose e halitofobia. A halitose genuína se divide em halitose fisiológica (causada principalmente pela saburra ligual) e halitose patológica que pode ser oral (doenças bucais) ou extra-oral (doenças sistêmicas). A pseudo-halitose consiste na autopercepção do paciente que relata a presença de mau hálito mesmo quando ele não é percebido por outras pessoas e não é diagnosticado clinicamente. Já a halitofobia é uma condição em que, mesmo sem evidências clínicas ou sociais, e após os tratamentos específicos, o paciente se queixa de mau hálito [2].

A prevalência da halitose é alta, sendo possível encontrar na literatura valores acima de 50% [3] e é considerada um importante fator social, pois interfere nas relações interpessoais. Além de gerar preocupações relacionadas à saúde física do indivíduo, pode provocar alterações psicológicas, conduzindo a uma barreira social [4]. Nesse contexto de importância social e biológica a prevalência e associações de halitose em populações pediátricas tem sido investigada no mundo com estimativas variadas [5-11].

Estudos recentes voltam um olhar atento às crianças respiradoras orais e demonstram que esse grupo apresenta aumento significativo do nível de halitose em comparação aos respiradores nasais [8, 12-14]. A halitose classificada como oral se origina na boca ou nas vias aéreas superiores e resulta da decomposição da matéria orgânica que origina-se de lascas de células epiteliais retidas no porção posterior do dorso da língua que ocorre, entre outros fatores, pela redução do fluxo salivar e/ou desequilíbrio hídrico e pelo ataque microbiano no meio bucal que favorecem o crescimento de bactérias proteolíticas e, consequentemente, resultam na produção de compostos voláteis de enxofre relacionados ao odor característico [15-16]. Quando a taxa de fluxo salivar diminui, a contagem bacteriana e a halitose na cavidade oral aumentam. A mudança da respiração nasal para respiração oral, causa adaptação, alterações nas arcadas dentárias e tecidos circundantes, como alterações anatômicas do palato e ressecamento da superfície da mucosa. A secagem superficial da mucosa em crianças respiradoras, que é uma das queixas principais em indivíduos que respiram pela boca, pode estar relacionada à halitose [3,17]. Pacientes com respiração oral por hipertrofia adenotonsilar avaliados

apresentaram maiores taxas de halitose quando comparado aos grupos de tratamento (cirurgia) e controle (respiradores nasais) [11].

Os Compostos Sulfurosos Voláteis (CSV) são componentes químicos que estão relacionados com a presença de halitose; o sulfidreto (relacionado à saburra lingual), o metilmercaptano (relacionado à bolsas periodontais) e o dimetilsulfeto (relacionado à alterações sistêmicas) [5, 18-20]. Existem diferentes métodos de diagnóstico de halitose: a avaliação clínica, conhecida como teste organoléptico, um método subjetivo que consiste em sentir o cheiro exalado pela boca e pelo nariz, e em seguida, quantificar esse odor com o uso de uma escala. Os CVS podem ser medidos com o uso de monitores de sulfeto e cromatografia gasosa [2,5,12,21]. O dispositivo portátil Breath-Alert (BA) tem sido cada vez mais empregado na prática clínica para o diagnóstico da halitose devido à sua facilidade de uso e baixo custo [17, 22-26]. Em crianças, que necessitam de exames rápidos e práticos, o BA é uma ferramenta para a detecção de halitose na prática da odontopediatria que demonstra alta sensibilidade e especificidade [8].

Os tratamentos convencionais utilizados no controle da halitose consistem basicamente no uso de dentifrícios e colutórios contendo substâncias bactericidas, uso de raspador lingual, tratamento das lesões de cárie e da doença periodontal, além do controle da xerostomia [27]. Alguns estudos sugerem que o fluoreto de amina tem efeito positivo na diminuição da halitose [3]. Estudos mostram que tratamentos alternativos, como a Terapia Fotodinâmica Antimicrobiana (aPDT) [11,22, 28] e probióticos, tem sido empregado na tentativa de controlar a halitose [3,17,23,24,27]. A aPDT é um tratamento no qual é utilizado um agente fotossensibilizador, um corante, que na presença de luz, produz radicais livres de oxigênio levando à morte celular, no caso da halitose que tem o principal fator etiológico relacionado com a presença de bactérias anaeróbias, essa terapia apresentou resultados positivos (com uso de laser vermelho e azul de metileno) na redução de sulfeto de hidrogênio, bem como na redução da carga bacteriana no dorso da língua [16,22]. O corante Urucum vem sendo avaliado como um fotossensibilizador em estudos relacionados a halitose. Extraído da semente da Bixa orellana, uma planta nativa do Brasil, urucum é aceito pela Organização Mundial da Saúde (OMS), devido ao fato de não ser tóxico [1]. Possui importante atividade antioxidante e antimicrobiana [10], e estudos recentes têm demonstrado seu potencial como agente terapêutico e corante natural [29].

Os probióticos são definidos como microrganismos que proporcionam efeitos benéficos para a saúde do hospedeiro quando absorvidos pelo mesmo. São

frequentemente utilizados em alimentos e produtos fermentados, além de serem utilizados em manipulações farmacêuticas [26]. As pesquisas mostram resultados positivos no uso de probióticos no controle da halitose e sugerem que eles possam favorecer a eliminação de alguns microrganismos indesejáveis e promover a recolonização da microbiota do indivíduo [17,23].

As vantagens de abordagens alternativas como aPDT e urucum e administração de probióticos para a redução ou eliminação da halitose na odontopediatria são técnicas menos invasivas que lançam uso de componentes naturais como urucum que pode reduzir os danos aos tecidos orais e evitar resistência bacteriana. É desafiador desenvolver, pesquisar estabelecer um protocolo de tratamento para halitose que possa ser eficaz, não traumático nessa faixa etária e duradouro, eliminando as bactérias anaeróbias relacionadas à essa condição e possivelmente pelo equilíbrio sistêmico, restabelecer a microbiota do dorso da língua, a fim de promover uma melhora na qualidade de vida integral do indivíduo. O uso de probióticos na odontologia apresenta um tratamento inovador, capaz de modificar a microbiota oral, como uma alternativa ao uso de antibióticos e outros produtos antimicrobianos.

O tratamento da halitose é um tema que ainda precisa de atenção e os resultados deste estudo podem apoiar a tomada de decisões dos profissionais de saúde em relação ao uso de probióticos e à aPDT usando LEDs azuis para tratar a halitose em seu cotidiano, já que a maioria dos dentistas já possui essa fonte de luz em seus consultórios e o medidor portátil é de baixo custo para aquisição. Além disso, a utilização do extrato urucum como fotossensibilizador é inovador. Uma vez que se trata de uma fonte de luz e um fotossensibilizador acessível, espera-se que este tratamento seja reproduzido clinicamente com eficácia e facilidade. Espera-se que o uso de probióticos e o uso da aPDT sejam eficazes na diminuição da halitose em crianças respiradoras orais.

2. Material e Métodos

2.1 Objetivo Geral

O objetivo do presente estudo é verificar se o tratamento com aPDT, utilizando extrato de urucum como fotossensibilizador e o LED azul como fonte de luz, é eficaz na redução em crianças respiradoras oral.

2.2 Objetivos específicos

- Verificar a alteração da halitose após o uso da terapia fotodinâmica empregando o uso de urucum como fotossensibilizador e LED imediatamente e após 7 dias.
- Avaliar alteração da halitose após o tratamento com probióticos por 7 dias de administração.
- Analisar a alteração microbiológica após a terapia fotodinâmica antimicrobiana.
- Analisar a alteração microbiológica após o tratamento com probióticos.

2.3 Delineamento Experimental

Tipo de Estudo: O presente trabalho se caracteriza como estudo ensaio clínico controlado e randomizado e será realizado nas dependências da Clínica Odontológica da Universidade Metropolitana de Santos (UNIMES). Por se tratar de um estudo clínico randomizado e buscando uma maior transparência e qualidade dessa pesquisa, utilizaremos as recomendações CONSORT (Consolidated Standards of ReportingTrials).

Hipótese

Hipótese nula:

- Não há alteração da halitose após o uso da terapia fotodinâmica empregando o uso de corante(fotossensibilizador) e LED azul.
- Não há alteração da halitose após o tratamento com probióticos
- Não há alteração microbiológica após a terapia fotodinâmica antimicrobiana. Não há alteração microbiológica após o tratamento com probióticos.

Hipótese experimental:

- Há diminuição da halitose após o uso da terapia fotodinâmica empregando o uso de corante urucum e LED azul.
- Há diminuição da halitose após o tratamento com probióticos.
- Há alteração microbiológica após a terapia fotodinâmica antimicrobiana.
- Há alteração microbiológica após o tratamento com probióticos.

2.4 Aspectos Éticos

O estudo será conduzido eticamente de acordo com os critérios descritos na Declaração de Helsinki (World Medical Association Declaration of Helsinki, 2008). O protocolo desse estudo será submetido à aprovação do Comitê de Ética em Pesquisa da Universidade Metropolitana de Santos (UNIMES). Todas as informações estarão presentes no Termo de consentimento livre e esclarecido (Resolução no. 196 do Conselho Nacional de Saúde, Ministério da Saúde, Distrito Federal, Brasil, 10/03/1996), os quais serão assinados em duas vias, pertencendo uma ao responsável, e outra aos pesquisadores.

Os participantes do estudo receberão também instruções de que poderão desistir do estudo a qualquer momento, por qualquer razão, se assim o desejarem. Os pesquisadores também poderão remover os participantes do estudo, caso achem necessário.

2.5 Determinação do tamanho da amostra

Para o cálculo do tamanho amostral foram utilizados os dados do trabalho de Costa da Mota et al. [22].

Inicialmente estabeleceu-se um erro $err= |\bar{x}_1 - \bar{x}_2|$, onde $\bar{x}_1$ e $\bar{x}_2$ sãos os valores médios dos grupos baseline a tratamento periodontal com PDT. A partir deste erro, foi calculado o effect size, dado por

$$\frac{err}{\sqrt{\sigma_1^2 + \sigma_2^2}}$$

onde σ_1^2 e σ_2^2 são as variâncias dos grupos um e dois, respectivamente.

Assumindo que os grupos estudados possuem distribuição normal ou aproximadamente normal, que o tamanho amostral será suficientemente grande e que será utilizado um teste bicaudal, para um nível de significância $\alpha = 0,05$ e mantendo o poder do teste $1-\beta = 0,90$, temos um n=13 para cada grupo.

Na figura 1 observa-se que com um tamanho amostral total de 52 sujeitos, ou seja, três grupos com 13 amostras cada, que deverá ser demonstrada a diferença estatística mantendo o poder do teste maior ou igual a 0.90. Caso a hipótese de normalidade das distribuições seja rejeitada, o tamanho amostral deverá ser corrigido em aproximadamente 5%.

Figura 1: Ajuste do poder do teste em função do tamanho amostral total.

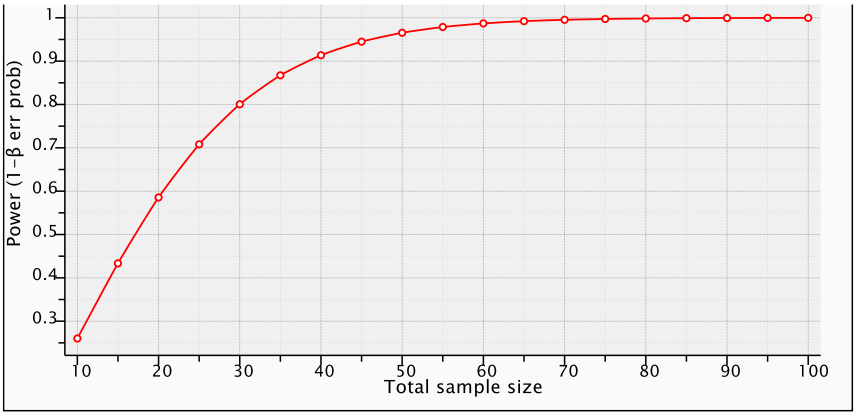

Serão selecionados 52 pacientes respiradores orais e com diagnóstico de halitose que serão divididos em 04 grupos.

2.6 População do estudo

Na primeira consulta um formulário contendo a história médica do paciente também será preenchido. Na sequência, esses voluntários serão submetidos a exame clínico, para a determinação das suas condições orais. Com base nas informações coletadas nessa primeira visita, o estudo seguirá os critérios de Inclusão e Exclusão.

2.6.1 Critérios de Inclusão

Serão incluídos nesta pesquisa participantes de ambos os sexos, de 7 a 12 anos, com diagnóstico de respiração oral, um questionário padronizado será usado para avaliar o hábito de respiração oral, e halitose apresentando no medidor portátil pontuação igual ou superior a 2.

Questões sobre renda dos pais, escolaridade, experiência anterior de tratamento odontológico dos filhos, presença de ronco, boca aberta durante o sono, sonolência diurna, sono agitado, saliva no travesseiro durante o sono, despertar com sede à noite, boca seca, enurese noturna, cefaleia , agressividade, desatenção, hiperatividade, respiração anormal, dificuldade de deglutição, olfato, paladar e fala, efeito negativo no

desempenho escolar e presença de alergias será questionados aos pais, e suas respostas serão registradas nas fichas de anamnese. Além disso, os pais serão questionados sobre a frequência de escovação de seus filhos, sua participação na escovação, hábitos alimentares da criança, creme dental da criança, presença de maus hábitos bucais como bruxismo, roer unhas, roer lábios, língua mordendo, empurrando, chupando os lábios, e se eles detectam mau hálito em seu filho ou em si mesmos.

2.6.2 Critérios de Exclusão

Serão excluídos do estudo indivíduos respiradores nasais, com anomalias dentofaciais (como lábio leporino, fissuras palatinas e nasopalatinas), em tratamento ortodôntico e/ou ortopédico, que estejam em tratamento oncológico, com alterações sistêmicas (gastrointestinais, renais, hepáticas), em tratamento com antibiótico até 1 mês antes da pesquisa, gestantes, língua fissurada ou sulcada.

2.7 Grupos de Estudo

Grupo 1: tratamento com escovação, fio dental e raspador de língua;
Grupo 2: escovação, fio dental e aPDT aplicada na região de dorso e terço médio da língua;
Grupo 3: escovação fio dental e probióticos;
Grupo 4: escovação, fio dental, aPDT e probióticos.

Serão comparados os resultados da halimetria antes, imediatamente após o tratamento, sete dias após o tratamento. Será realizada a análise microbiológica da saburra lingual nestes mesmos tempos. A análise quantitativa será realizada por técnica de plaqueamento direto e PCR em tempo real.

2.8 Randomização

O tipo de tratamento será determinado aleatoriamente para cada paciente, através da realização de um sorteio antes da intervenção. O sorteio seguirá ordem gerada eletronicamente pelo site de randomização randomizer.org para distribuição de maneira equilibrada de todos os dentes entre os grupos.

2.9 Intervenções

Por se tratar de um estudo clínico randomizado e buscando uma maior transparência e qualidade dessa pesquisa, utilizaremos as recomendações CONSORT (Consolidated Standards of Reporting Trials) (Figura 2).

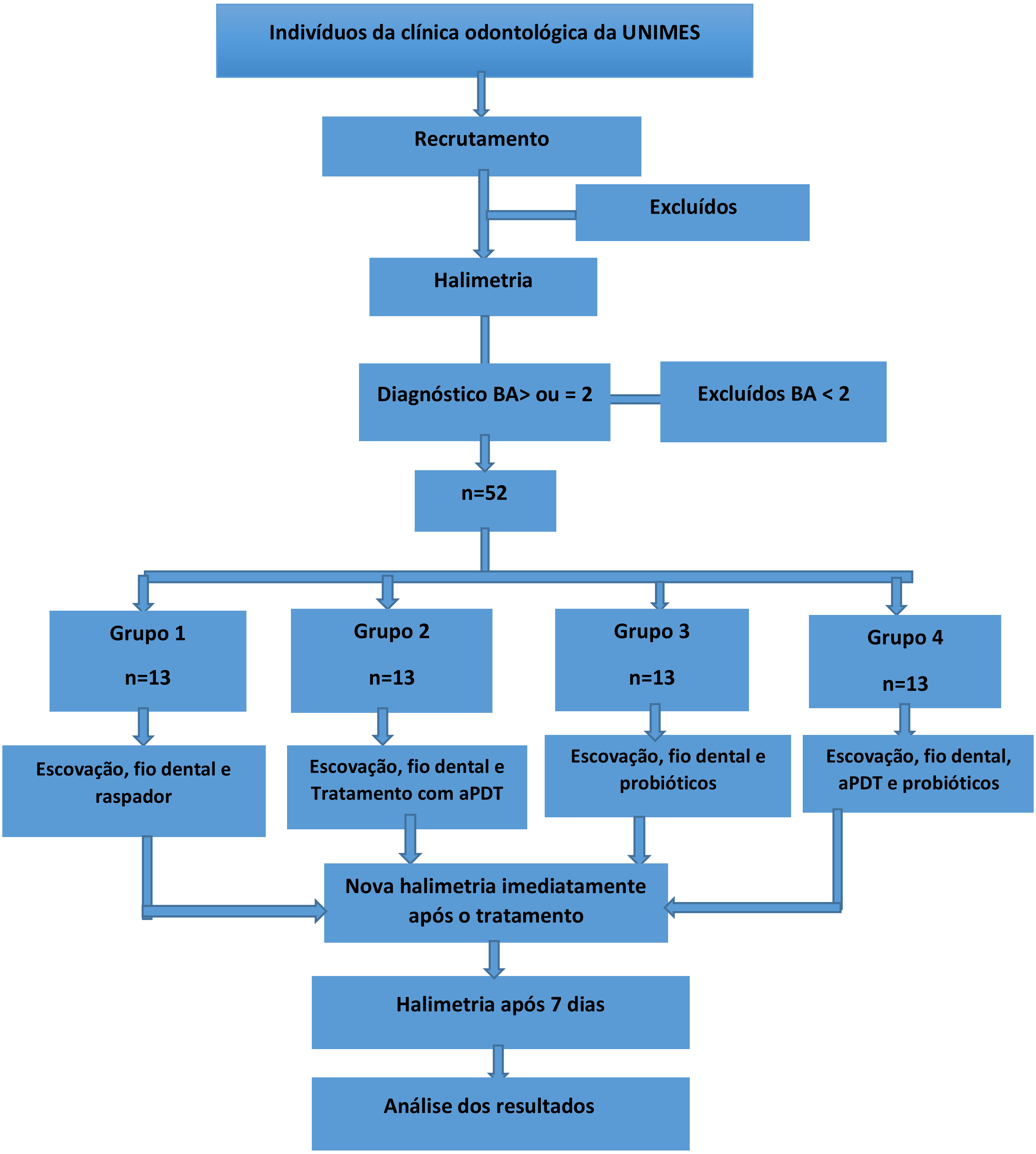

Figura 2: Fluxograma de atividades.

Avaliação da Saburra Lingual

A quantidade de Saburra lingual será definida pelo Índice de Saburra Lingual (CTI – Coated Tongue Index) proposto por Shimizu et al. [30] A língua é dividida em 9 partes, para cada parte é dada uma nota, sendo 0 – ausência de saburra lingual , 1 – presença de saburra lingual com papilas visiveis, 2 – espessura de Recrutamento Incluídos: SH2>112 ppb Excluídos: SH2

Halimetria

A coleta do ar bucal seguirá as orientações do fabricante o BA será desinfetado após cada uso. O aparelho deve ser sacudido quatro ou cinco vezes antes de cada uso para eliminar quaisquer odores residuais. Um ‘‘bip’’ é emitido ao abrir o compartimento superior do o dispositivo, e um segundo ‘‘bipe’’ é emitido quando o voluntário sopra na entrada de ar frontal (passagem do fluxo de ar). Após um terceiro “bip”, o odor do hálito é medido e pontuado em uma escala de 0 a 5 pontos. Quando a letra ‘‘E’’ aparece

indica erro, o procedimento é repetido (Figura 3).

Um escore > ou = 2 é considerado indicativo de halitose.

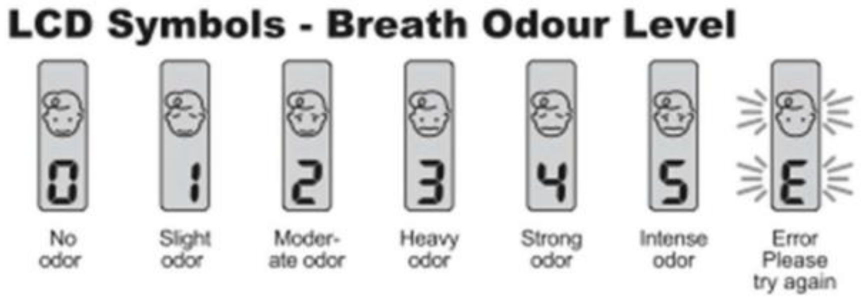

Figura 3: Processo de realização da halimetria.

Para evitar alterações na halimetria os participantes serão instruídos a seguir as seguintes orientações: 48 horas antes da avaliação evitar a ingestão de alimentos com alho, cebola e temperos fortes, consumo de álcool e uso de antisséptico bucal. No dia da avaliação, pela manhã, poderão alimentar-se até no máximo 2 horas antes do exame, abster-se de café, balas, goma de mascar, produtos de higiene oral e pessoal com perfume (pós-barba, desodorante, perfume, cremes e/ou tônico) e a escovação será apenas com água.

Análise Microbiológica

As coletas microbiológicas serão realizadas antes e imediatamente após o tratamento. Amostras da saburra lingual serão coletadas com um cotonete, embebido em carvão Amies reduzido como meio.

As amostras serão levadas para o laboratório para análise em período inferior a duas horas após a coleta. As amostras serão colocadas em vórtex por aproximadamente um minuto. Após a homogeneização, dez diluições seriadas foram preparadas em 180 µl de solução salina tamponada com fosfato estéril. Alíquotas de 10-1, 10–2, 10–3, 10–4 e 10–5 serão transferidos para placas de Petri com ágar sangue de ovelha. Como as principais bactérias responsáveis pela produção de CSVs são anaeróbios, as placas de Petri serão incubadas em anaerobiose por 72 h a 37°C, a seguir as contagens bacterianas serão realizadas por UFCs [4,22].

Análise Microbiológica PCR Tempo real

As amostras da saburra lingual serão coletadas utilizando 1 swab estéril que será passado na superfície do dorso de língua, realizando 1 movimento de vai e vem para a coleta de saburra lingual. As amostras serão depositadas em tubos estéreis contendo Tris – EDTA buffer (10mM Tris – HCL, o,1mM EDTA, pH7,5) que serão identificados e

armazenados à -80 C até serem analisados (o tempo máximo de armazenamento será de 1 semana). As amostras serão congeladas devido à impossibilidade de realizar todas as análises em um único dia [3]. Após descongelamento, as amostras serão submetidas ao vórtex por um minuto. Para extração do DNA bacteriano será utilizado o Kit Puro Master DNA Extraction (Epicentre Tecnologies, Chigago, Illinois, USA). Para iniciar a extração utilizar-se á 100µL de amostra eluída em TE. Serão centrifugadas a 10.000 rpm a 4º C por 10 minutos. Na sequência será preparado o MIX (diluir 300µl TCL + 2 µl Prot K para cada amostra). Será retirado o sobrenadante com pipeta e sobrará 25µl de volume + o pellet formado. Vortex por 10s para ressuspender o pellet. Será adicionado 302 µl de MIX em cada amostra e vortex por 10s. será incubado a 65º C por 15 min e vortex a cada 5 min. As amostras serão colocadas no gelo por 5 min. Será adicionado 150 µl de MPC (protein precipitation reagente) às amostras e vórtex 10 segundos. Será centrifugado a 10.000 rpm a 4 o C por 10 minutos. Após centrifugar tirar todo o sobrenadante será colocado no tubo novo. Joga-se fora o tubo com pellet. Adiciona-se 25 µl de MPC (protein precipitation) ao sobrenadante (já colocado no tubo novo). Centrifuga-se a 10.000 rpm a 4 o C por 10 minutos. Adiciona-se 500 µl de isopropanol ao sobrenadante e inverte 40x. Centrifuga-se a 10.000 rpm a 4 o C por 10 minutos. Tirar amostras da centrifuga e deixar no gelo, para não soltar o pellet. Verte o isopropanol e tira-se restante com pipeta de 50 µl. Lava-se 200 µl de etanol 70% cuidadosamente para não desalojar o pellet. Centrifugar a 10.000 rpm a 4 oC por 3 minutos. Remover todo etanol residual. Lavar 200 µl de etanol 70% cuidadosamente para não desalojar o pellet. Centrifugar a 10.000 rpm a 4 o C por 3 minutos. Remove-se todo etanol residual. Deixa 30min invertidos secando. Eluir em 40 µl de TE . Congelar a -20 [32]. O DNA purificado será ressuspenso em tampão TE. Serão analisados os níveis de P. gingivalis, T. forsythia e T. denticola, por PCR quantitativo. A análise quantitativa será realizada por meio de PCR

em tempo real utilizando termociclador Step One Plus Real-Time PCR System (Applied Biosystem, Foster City, CA, EUA) e os produtos detectados por fluorescência usando o Quantimix Easy SYG Kit (Biotools, Madri, Espanha), seguindo o protocolo recomendado pelo fabricante. Para a reação serão utilizados 10 µl de SYBR Green, 0,5 µl DNA molde, 200 mM de cada iniciador (P.gingivalis CATAGATATCACGAGGAACTCCGA TT e AAACTGTTAGCAACTACCGATGTGG; T.forsythia GGGTGAGTAACGCGTATGTAACCT e ACCCATCCGCAACCAATAAA; T. denticola CGTTCCTGGGCCTTGTACA e TAGCGACTTCAGGTACCCTCG; Universal para bactéria CCATGAAGTCGGAATCGCTAG e GCTTGACGGGCGGTGT) em volume total de 20 µl. Como controle negativo será adicionada água milliQ estéril ao invés de DNA molde. As reações para 16S rRNA serão realizadas com desnaturação inicial de 95 ºC por 2 minutos, seguida de 36 ciclos de 94 ºC por 30 segundos, 55 ºC por 1 minuto e 72 ºC por 2 minutos e extensão final a 72 ºC por 10 minutos. A fluorescência será detectada após cada ciclo e representada em um gráfico utilizando o software Step One Plus Real- Time PCR System (Applied Biosystem, Foster City, CA, EUA). Para garantir a especificidade dos produtos detectados por fluorescência e evitar a detecção de dímeros de iniciadores, a detecção será realizada um grau abaixo da temperatura de dissociação dos amplicons. Todas as amostras serão analisadas em duplicata e cada diluição dos plasmídeos para a curva padrão em triplicata. A finalidade da avaliação microbiológica será verificar a efetividade da terapia fotodinâmica para o tratamento da halitose, complementando a avaliação clínica.

Terapia Fotodinâmica Antimicrobiana (aPDT)

Será utilizado o aparelho fotopolimerizador LED – Valo Cordless Ultradent®, um aparelho de consultório, com radiômetro acoplado, espectro de 440-480nm e irradiância de 450mW/cm. No momento da realização da aPDT estarão presentes somente o

voluntário a ser tratado e o profissional responsável, ambos utilizando óculos específicos para proteção ocular. A ponta ativa do LED será revestida com plástico transparente descartável (PVC) (evitando contaminação cruzada e por motivo de higiene) e o profissional estará devidamente paramentado.

Será realizada 1 sessão de aPDT com o fotossensibilizador (FS) urucum manipulado na concentração 20% (Fórmula e Ação®) em spray, a ser aplicado em quantidade suficiente para cobrir o terço médio e dorso da língua (5 borrifadas) por 2 minutos para incubação. O excesso, se houver, será removido com sugador de forma a manter a superfície úmida com o próprio FS, sem utilização de água. Serão irradiados 6 pontos com distância de 1 cm entre os pontos, considerando o halo de espalhamento da luz e efetividade da aPDT. O aparelho estará previamente calibrado com comprimento de onda 395-480 nm, durante 20 segundos por ponto, energia de 9,6J, e a luz era irradiada de modo que seja formado um halo de 2 cm de diâmetro por ponto. A Tabela 1 contém todos os parâmetros utilizados. [31].

Tabela 1: parâmetros do LED.

**Comprimento de onda (nm)**	395-480
**Modo de funcionamento**	Contínuo
**Potência radiante média (mW)**	480
**Polarização**	aleatória
**Diâmetro de abertura (cm)**	0.9
**Irradiância na abertura (mW/cm2)**	762
**Perfil do Feixe**	Top hat
**Área irradiada (cm2)**	3.14
**Irradiância no alvo (mW/cm2)**	153
**Tempo de exposição (s)**	20

**Fluência (J/cm2)**	6.37
**Energia radiante (J)**	9.6
**Número de pontos irradiados**	6
**Área total irradiada (cm2)**	18.8
**Número de sessões**	1
**Energia radiante total (J)**	57.6

Raspagem Lingual

A raspagem lingual será realizada por um mesmo operador em todos os participantes. Serão realizados movimentos póstero-anteriores com o raspador sobre o dorso lingual, seguidos da limpeza do raspador com uma gaze. Esse procedimento será realizado dez vezes em cada paciente, com o objetivo de padronizar a remoção mecânica da saburra lingual.

Tratamento com probióticos

Serão utilizadas formas farmacêuticas (cápsulas ou gomas mastigáveis manipuladas em farmácia contendo cepas de Lactobacillus salivarius WB21 (6,7 x 10^8 UFC) e xilitol (280mg). Serão entregues para cada paciente, que deverá ingerir, 3 vezes ao dia após as refeições, durante 7 dias.

Escovação com dentifrício com fluoreto de amina

Todos os 52 participantes serão orientados a realizar escovação com dentifrício contendo fluoreto de amina em sua composição (Elmex®) e uso de fio dental, 3 vezes ao dia após as refeições durante 10 dias.

Organização e Tratamento Estatístico dos Dados

Os dados oriundos do medidor portátil BA™ serão analisados quanto à sua normalidade pelo teste de Shapiro – Wilk. Caso a hipótese de normalidade seja aceita, será utilizada a Análise de variância (ANOVA) seguida pelo teste de Tukey quando necessário. Para analisar os resultados do tratamento nos dois períodos do estudo será utilizado o teste T para dados pareados. Caso a hipótese de normalidade seja rejeitada, será utilizado o teste Kruskal-Wallis seguido pelo teste de Student-Newman-Keuls, quando necessário. Para analisar os resultados de cada tratamento nos dois períodos do estudo será utilizado o teste de Wilcoxon.

3. Resultados Esperados

Por meio do presente trabalho poderemos avaliar, se haverá diferença de efetividade entre os tratamentos propostos, bem como se haverá diminuição da halitose após o uso da terapia fotodinâmica empregando o uso de corante urucum e LED azul e após o tratamento com probióticos.

4. Cronograma de Execução

**Atividades**	Pré intervenção			1	2	3	4	5	6	7	8	9	10
Submissão Comitê de Ética em Pesquisa													
Seleção dos participantes e randomização													
Procedimentos													
Coleta dos dados													
Análise dos dados													
Redação e apresentação dos resultados obtidos													
Submissão dos artigos científicos													
Relatório final													

5. Referências

1. BAWAZIR O. A. (2021). Risk Factors, Diagnosis, and Management of Halitosis in Children: A Comprehensive Review. The journal of contemporary dental practice, 22(8), 959–963.
2. PORTER, S. R.; SCULLY, C. Oral malodour (halitosis). Bmj, v. 333, n. 7569, p. 632-635, 2006.
3. BICAK, Damla Aksit. A current approach to halitosis and oral malodor-A mini review. The open dentistry journal, v. 12, p. 322, 2018.
4. ELIAS, Marina Sá; FERRIANI, Maria das Graças Carvalho. Aspectos históricos e sociais da halitose. Revista Latino-Americana de Enfermagem, v. 14, n. 5, 2006.
5. Guedes, C. C., Bussadori, S. K., Garcia, A., Motta, L. J., Gomes, A. O., Weber, R., & Amancio, O. (2020). Accuracy of a portable breath meter test for the detection of halitosis in children and adolescents. Clinics (Sao Paulo, Brazil), 75, e1764. https://doi.org/10.6061/clinics/2020/e1764

6. AlMadhi, N.A.; Sulimany, A.M.; Alzoman, H.A.; Bawazir, O.A. Halitosis in Children Undergoing Full Mouth Rehabilitation under General Anesthesia. Children 2021, 8, 149. https://doi.org/10.3390/children8020149.
7. Małgorzata Sikorska-Żuk and Marek Bochnia Halitosis in children with adenoid hypertrophy, 2018 J. Breath Res. 12 026011.
8. Alqutami, J., Elger, W., Grafe, N., Hiemisch, A., Kiess, W., & Hirsch, C. (2019). Dental health, halitosis and mouth breathing in 10-to-15 year old children: A potential connection. European journal of paediatric dentistry, 20(4), 274–279. https://doi.org/10.23804/ejpd.2019.20.04.03.
9. Bawazir O. A. (2021). Risk Factors, Diagnosis, and Management of Halitosis in Children: A Comprehensive Review. The journal of contemporary dental practice, 22(8), 959–963.
10. Motta LJ, Bachiega JC, Guedes CC, Laranja LT, Bussadori SK. Association between halitosis and mouth breathing in children. Clinics (Sao Paulo). 2011;66(6):939-942. doi:10.1590/s1807-59322011000600003.
11. İnönü-Sakallı N, Sakallı C, Tosun Ö, Akşit-Bıçak D. Comparative Evaluation of the Effects of Adenotonsillar Hypertrophy on Oral Health in Children. Biomed Res Int. 2021;2021:5550267. Published 2021 Apr 2. doi:10.1155/2021/5550267
12. KARA, C; TEZEL, A; ORBAK, R. Effect of oral hygiene instruction and scaling on oral malodour in a population of Turkish children with gingival inflammation. Int J Paediatr Dent, v. 16, n. 6, p. 399–404, nov. 2006.
13. Eli I, Koriat H, Baht R Rosenberg M. Self-perception of breath odor: role of body image and psychopathologic traits. Percept Mot Skills. 2000;91 (3 pt 2):1193-201, doi: 10.2466/PMS.91.7.1193-1201. 9. Yaegaki K, Coil JM. Examination, classification, and treatment of halitosis; clinical perspectives. J Can Dent Assoc. 2000;66:257-61. 10.
14. Riggio MP, Lennon A, Rolph HJ, Hodge PJ, Donaldson A, Maxwell AJ, et al. Molecular identification of bacteria on the tongue dorsum of subjects with and without halitosis. Oral Dis. 2008;14:251-8, doi: 10.1111/ j.1601-0825.2007.01371.x.
15. TOLENTINO, E. D. S.; CHINELLATO, L. E. M.; TARZIA, O. Saliva and tongue coating pH before and after use of mouthwashes and relationship with parameters of halitosis. J Appl Oral Sci, v. 19, n. 2, p. 90–4, abr. 2011.

16. DA CIARCIA, Ana Carolina Costa et al. Action of antimicrobial photodynamic therapy with red leds in microorganisms related to halitose. Medicine, v. 98, n. 1, 2019.
17. YOO, Jun-Il et al. The Effect of Probiotics on Halitosis: a Systematic Review and Meta-analysis. Probiotics and antimicrobial proteins, v. 11, n. 1, p. 150-157, 2019.
18. CALIL, CM.; MARCONDES, FK. Influence of anxiety on the production of oral volatile sulfur compounds. Life Science, v. 79, n. 7, p. 660–4, 10 jul. 2006.
19. SPRINGFIELD, J. et al. Spontaneous fluctuations in the concentrations of oral sulfurcontaining gases. J Dental Res, v. 80, n. 5, p. 1441–1444, 2001.
20. TANGERMAN, A; WINKEL, E. G. The portable gas chromatograph OralchromaTM: a method of choice to detect oral and extra-oral halitosis. J Breath Res, v. 2, n. 1, mar. 2008.
21. KARA, C. et al. Effect of Nd: YAG laser irradiation on the treatment of oral malodour associated with chronic periodontitis. Int Dent J, v. 58, p. 151–158, 2008.
22. COSTA DA MOTA, Ana Carolina et al. Effect of photodynamic therapy for the treatment of halitosis in adolescents–a controlled, microbiological, clinical trial. Journal of biophotonics, v. 9, n. 11-12, p. 1337-1343, 2016.
23. López-Valverde N, López-Valverde A, Macedo de Sousa B, Rodríguez C, Suárez A and Aragoneses JM (2022) Role of Probiotics in Halitosis of Oral Origin: A Systematic Review and Meta-Analysis of Random
24. Motta, P. B., Motta, L. J., Campos, T. M., Gonçalves, M., Santos, E. M., Martimbianco, A., de Andrade, D., Mesquita-Ferrari, R. A., Fernandes, K., Horliana, A., & Bussadori, S. K. (2022). Effect of Photodynamic Therapy on Halitosis: A Systematic Review of Randomized Controlled Trials. Sensors (Basel, Switzerland), 22(2), 469. https://doi.org/10.3390/s22020469
25. HOPE, Chris K.; WILSON, M. Induction of lethal photosensitization in biofilms using a confocal scanning laser as the excitation source. Journal of Antimicrobial Chemotherapy, v. 57, n. 6, p. 1227-1230, 2006.
26. SALMINEN, Seppo et al. Demonstration of safety of probiotics—a review. International journal of food microbiology, v. 44, n. 1-2, p. 93-106, 1998.
27. SUZUKI, Nao et al. Induction and Inhibition of Oral Malodor. Molecular Oral Microbiology, 2019.
28. KELLESARIAN, Sergio Varela et al. Effect of antimicrobial photodynamic therapy and laser alone as adjunct to mechanical debridement in the management of halitosis: A systematic review. Quintessence International, v. 48, n. 7, 2017.
29. Vilar Daniela de Araújo, Vilar Marina Suênia de Araujo, Moura Túlio Flávio Accioly de Lima e, Raffin Fernanda Nervo, Oliveira Márcia Rosa de, Franco Camilo Flamarion de Oliveira, de Athayde-Filho Petrônio Filgueiras, Diniz Margareth de Fátima Formiga

Melo, Barbosa-Filho José Maria. Traditional Uses, Chemical Constituents, and Biological Activities ofBixa orellanaL.: A Review. The Scientific World Journal. 2014;2014:1–11. doi: 10.1155/2014/857292.

30. Shimizu T, Ueda T and Sakurai K 2007 New method for evaluation of tongue-coating status J. Oral Rehabil. 34 442–.
31. GONÇALVES, Marcela Leticia Leal et al. Photodynamic therapy with Bixa orellana extract and LED for the reduction of halitosis: study protocol for a randomized, microbiological and clinical trial. Trials, v. 19, n. 1, p. 590, 2018.
