# Supplementary material for: Assessment of photodynamic therapy with annatto and led for the treatment of halitosis in mouth-breathing children: Randomized controlled clinical trial
Source: PLoS One. 2024 Sep 3;19(9):e0307957. doi: 10.1371/journal.pone.0307957 (PMC11371243; doi:10.1371/journal.pone.0307957)
Supplement: S5 File — (PDF) [file pone.0307957.s006.pdf]

RESEARCH PROJECT

COMPARATIVE STUDY OF PHOTODYNAMIC THERAPY AND PROBIOTICS FOR THE REDUCTION OF HALITOSIS IN MOUTH-BREATHING CHILDREN: PROTOCOL FOR A RANDOMIZED CONTROLLED CLINICAL TRIAL

Head researcher: Prof. Dr. Ana Paula Taboada Sobral

Santos
2022

ABSTRACT

Halitosis is a term that defines any odor or foul smell the emanates from the oral cavity, the origin of which may be local or systemic. One of the causes of local or oral halitosis is low salivary flow and dry mouth, which is also one of the complaints of individuals with the mouth-breathing habit. The aim of this project is to determine the effectiveness of antimicrobial photodynamic therapy (aPDT) and the use of probiotics for the treatment of halitosis in mouth-breathing children. Fifty-two children between seven and 12 years of age with a diagnosis of mouth breathing and halitosis determined through an interview and clinical examination will be selected. The participants will be divided into four groups: Group 1 – treatment with brushing, dental floss and tongue scraper; Group 2 – brushing, dental floss and aPDT applied to the dorsum and middle third of the tongue; Group 3 – brushing, dental floss and probiotics; Group 4 – brushing, dental floss, aPDT and probiotics. The use of a breath meter and microbiological analysis of the tongue coating will be performed before, immediately after treatment and seven days after treatment. The quantitative analysis will involve counts of colony-forming bacteria per milliliter and real-time PCR. The normality of the data will be determined using the Shapiro–Wilk test. Parametric data will be submitted to analysis of variance (ANOVA) and nonparametric data will be compared using the Kruskal–Wallis test. The results of each treatment in the different periods of the study will be compared using the Wilcoxon test.

Keywords: Halitosis, Photodynamic Therapy, *Bixa orellana*, Probiotics

1. Introduction

Halitosis is a term that defines any odor that emanates from the oral cavity, the origin of which may be local or systemic [1]. The odor may be attributed to a variety of products resulting from the metabolism of bacterial amino acids. Halitosis is classified as genuine, pseudo-halitosis and halitophobia. Genuine halitosis is divided into physiological halitosis (caused mainly by coated tongue) and pathological halitosis, which can be oral (oral disease) or extraoral (systemic diseases). Pseudo-halitosis is self-perceived by the individual even when not perceived by others and not diagnosed clinically. Halitophobia is a condition in which the patient complains of bad breath even with no clinical or social evidence and after specific treatments [2].

The prevalence of halitosis is high, with the literature reporting rates of more than 50% [3]. This condition is considered an important social factor that interferes with interpersonal relationships. Besides causing concerns related to physical health, halitosis can be a social barrier and lead to psychological problems [4]. In this context of social and biological importance, the prevalence of halitosis and associated factors in the pediatric population has been investigated throughout the world and different rates are estimated [5-11].

Recent studies involving mouth-breathing children have demonstrated that this group has a significantly greater frequency of halitosis compared to nose breathers [8, 12-14]. Halitosis classified as oral originates in the mouth and upper airways and results from the decomposition of organic matter – epithelial cells are retained in the posterior portion of the tongue due to low salivary flow and/or a water imbalance as well as microbial attack in the oral environment, which favor the growth of proteolytic bacteria, resulting in the production of volatile sulfur compounds, which are related to the characteristic odor [15-16]. When salivary flow rate is low, the bacterial count and halitosis in the oral cavity increase. The change from nose breathing to mouth breathing causes changes in the dental arches and surrounding tissues, such as anatomic changes in the palate and the drying of the surface of the mucosa, which is one of the main complaints of mouth breathers and may be related to halitosis [3,17]. Individuals who breath through the mouth due to adenotonsillar hypertrophy have higher rates of halitosis compared to treatment groups submitted to surgery and control groups (nose breathers) [11].

Volatile sulfur compounds (VSCs) are related to the occurrence of halitosis. Hydrogen sulfide is associated with coated tongue, methyl mercaptan is associated with periodontal pockets and dimethyl sulfide is associated with systemic alterations [5, 18-20]. Among the different methods for the diagnosis of halitosis, the clinical

examination, known as the organoleptic test, is a subjective method consisting of smelling the odor exhaled from the mouth and nose and quantifying this odor with the use of a scale. VSCs can be measured with the use of sulfide monitors and gas chromatography [2,5,12,21]. The portable Breath-Alert device (BA) has been used with increasing frequency in clinical practice for the diagnosis of halitosis due to its ease of use and low cost [17, 22-26]. In children, who need fast, practical exams, the Breath-Alert device is a halitosis detection tool with high sensitivity and specificity for use in pediatric dentistry [8].

Conventional treatments used in the control of halitosis consist basically of the use of toothpastes and mouthwashes containing bactericidal substances, a tongue scraper, the treatment of dental caries and periodontal disease and the control of xerostomia [27]. Some studies suggest that amine fluoride has a positive effect on diminishing halitosis [3]. Alternative treatments, such as antimicrobial photodynamic therapy (aPDT) [11,22, 28] and probiotics, have also been employed in an attempt to control this condition [3,17,23,24,27]. Antimicrobial PDT is treatment in which a photosensitizing agent (dye) produces oxygen free radicals in the presence of light, leading to bacterial cell death. As the main etiological factor of halitosis is the presence of anaerobic bacteria, aPDT (red laser and methylene blue) has demonstrated positive results on the reduction of hydrogen sulfide as well as the bacterial load on the dorsum of the tongue [16,22]. The dye annatto has been evaluated as a photosensitizer in studies on halitosis. This extract from the seeds of the plant *Bixa orellana*, which is native to Brazil, is accepted by the World Health Organization due to the fact that it is non-toxic [1]. Annatto has antioxidant and antimicrobial properties [10] and recent studies have demonstrated its potential as a therapeutic agent and natural dye [29].

Probiotics are microorganisms that provide health benefits when absorbed by the host and are often used in foods and fermented products as well as pharmaceutical formulations [26]. Studies have shown the positive results of probiotics for the control of halitosis and suggest that these products favor the elimination of undesirable microorganisms and promote the recolonization of the microbiota [17,23].

Alternatives such as aPDT with annatto and the administration of probiotics for the reduction in or elimination of halitosis in pediatric dentistry are less invasive methods that involve natural components, which reduces harm to oral tissues and avoids the occurrence of bacterial resistance. The major challenge is the development of an effective, lasting treatment protocol for halitosis in children that eliminates the anaerobic bacteria related to this condition and reestablishes the microbiota of the dorsum of the tongue, thereby improving quality of life. The use of probiotics in dentistry constitutes innovative treatment capable of modifying the oral microbiota as an

alternative to the use of antibiotics and other antimicrobial agents.

The treatment of halitosis is a topic that requires greater attention and the results of this study can assist in clinical decision making with regards to the use of probiotics and aPDT with blue light-emitting diodes (LEDs) for the treatment of this condition, as most dentists have this light source in their offices and the portable sulfide meter is inexpensive to acquire. Moreover, the use of annatto as a photosensitizer is an innovative approach. As the light source and photosensitizer are accessible, this treatment is expected to be easily and effectively reproducible clinical practice. Probiotics and aPDT are expected to be effective at diminishing halitosis in mouth-breathing children.

2. Materials and Methods

2.1 General objective

The objective of the present study is to determine whether treatment with aPDT using annatto as the photosensitizer and blue led as the light source is effective at diminishing halitosis in mouth-breathing children.

2.2 Specific objectives

- Determine the change in halitosis after the use of photodynamic therapy employing annatto as a photosensitizer and LED immediately and after seven days.
- Assess the change in halitosis after treatment with probiotics administered for seven days.
- Analyze the microbiological change after antimicrobial photodynamic therapy.
- Analyze the microbiological change after treatment with probiotics.

2.3 Experimental design

<u>Type of study:</u> The present study is characterized as a randomized controlled clinical trial and will be conducted at the dental clinic of *Universidade Metropolitana de Santos* (UNIMES). The Consolidated Standards of Reporting Trials (CONSORT statement) will be followed to ensure greater transparency and the quality of the randomized clinical trial.

<u>Hypotheses</u>

<u>Null hypotheses:</u>

- There is no change in halitosis after the use of photodynamic therapy involving the annatto dye as the photosensitizer and blue LED as the light source.
- There is no change in halitosis after treatment with probiotics.
- There is no microbiological change after antimicrobial photodynamic therapy.
- There is no microbiological change after treatment with probiotics.

<u>Experimental hypotheses:</u>

- There is a reduction in halitosis after the use of photodynamic therapy involving the annatto dye as the photosensitizer and blue LED as the light source.
- There is a reduction in halitosis after treatment with probiotics.
- There is a microbiological change after antimicrobial photodynamic therapy.
- There is a microbiological change after treatment with probiotics.

2.4 Ethical aspects

The study will be conducted in accordance with the ethical precepts stipulated in the Declaration of Helsinki (World Medical Association Declaration of Helsinki, 2008). The protocol for this study will be submitted for approval of the Human Research Ethics Committee of *Universidade Metropolitana de Santos* (UNIMES). All information will be contained in the statement of informed consent in accordance with Resolution 196 of the National Board of Health (Health Ministry, Federal District, Brazil, March 10, 1996), two copies of which will be signed – one for the guardian and one for the researchers.

The participants will be informed that they may withdraw from the study at any time for any reason, if they so wish. The researchers will also be able to remove participants from the study if deemed necessary.

2.5 Determination of sample size

The sample size will be calculated using data from the study by Costa da Mota et al. [22]. An error = |(x_1) ̄-(x_2) ̄| was first established, in which (x_1) ̄ and (x_2) ̄are the means of the groups at baseline for periodontal treatment with PDT. The effect size was calculated based on this error as follows:

$$\frac{err}{\sqrt{\sigma_1^2 + \sigma_2^2}}$$

in which σ_1^2 and σ_2^2 are the variances of Groups 1 and 2, respectively.

Assuming that the groups studied have normal or approximately normal distribution, that the sample size will be sufficiently large and that a two-tailed test will be used for a significance level of $\alpha = 0.05$ and maintaining a test power of $1-\beta = 0.90$, $n = 13$ will be needed for each group.

Figure 1 shows that a sample of 52 children (four groups of 13 individuals) would enable demonstrating statistically significant differences while maintaining a test power equal to or higher than 0.90. If the hypothesis of normality of the distributions is rejected, the sample size should be corrected by approximately 5%.

Figure 1: Adjustment of test power as function of total sample size.

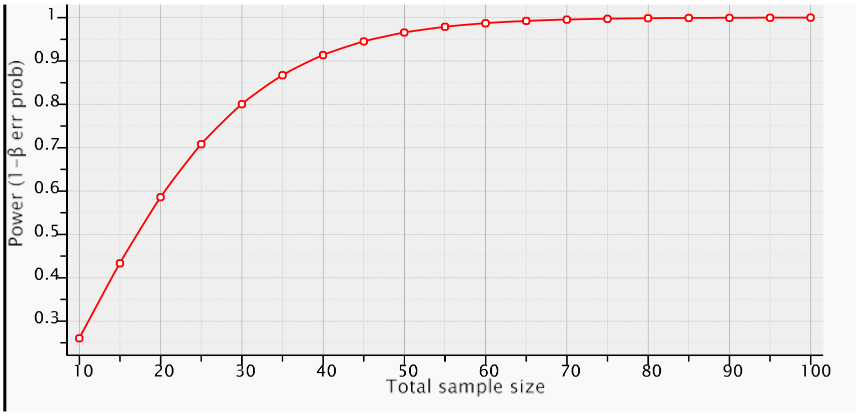

2.6 Study population

At the first appointment, a form addressing the medical history of the patient will be completed. The volunteers will then be submitted to a clinical examination for the determination of oral health status. Next, the inclusion and exclusion criteria will be applied.

2.6.1 Inclusion criteria

Male and female children seven to 12 years of age with a diagnosis of mouth breathing determined using a standard questionnaire and halitosis determined using a portable breath meter (score ≥ 2) will be included in the study.

Parents/guardians will provide data on family income, parent's schooling, child's previous experience with dental treatment, the occurrence of snoring, open mouth during sleep, daytime sleepiness, saliva on the pillow during sleep, awaking with thirst during the night, dry mouth, nocturnal enuresis, headache, aggressivity, inattention, hyperactivity, abnormal respiration, swallowing difficulty, sense of smell and taste, speaking, negative effect on school performance and the presence of allergies. Parents/guardians will also report their child's toothbrushing frequency, their participation in brushing, the child's eating habits, child's toothpaste, the occurrence of poor oral habits, such as bruxism, nail biting, lip biting, tongue bite, tongue pushing, smacking the lips and whether they have detected bad breath in their child or themselves.

2.6.2 Exclusion criteria

Nose breathers, individuals with dentofacial anomalies (e.g., hare lip and cleft palate), those in orthodontic and/or orthopedic treatment, those in oncological treatment, those with systemic (gastrointestinal, renal or hepatic) conditions, pregnant girls, individuals having undergone antibiotic treatment in the previous month and individuals with fissured tongue will be excluded.

2.7 Study groups

Fifty-two mouth-breathing children with a diagnosis of halitosis will be selected and divided into four groups.

Group 1: Treatment with brushing, dental floss and tongue scraper;
Group 2: Brushing, dental floss and aPDT applied to the dorsum and middle third of the tongue;
Group 3: Brushing, dental floss and probiotics;
Group 4: Brushing, dental floss, aPDT and probiotics.

Breath meter results will be determined before, immediately after treatment and seven days after treatment. Microbiological analysis of the tongue coating will performed at the same times. The quantitative analysis will be performed using direct plating and real-time PCR.

2.8 Randomization

The type of treatment will be determined randomly for each patient prior to the intervention. Randomization will be generated electronically using the randomizer.org site for the balanced distribution of the children among the groups.

2.9 Interventions

As a randomized clinical trial, the Consolidated Standards of Reporting Trials (CONSORT statement) will be followed to ensure greater transparency and the quality of the study (Figure 2).

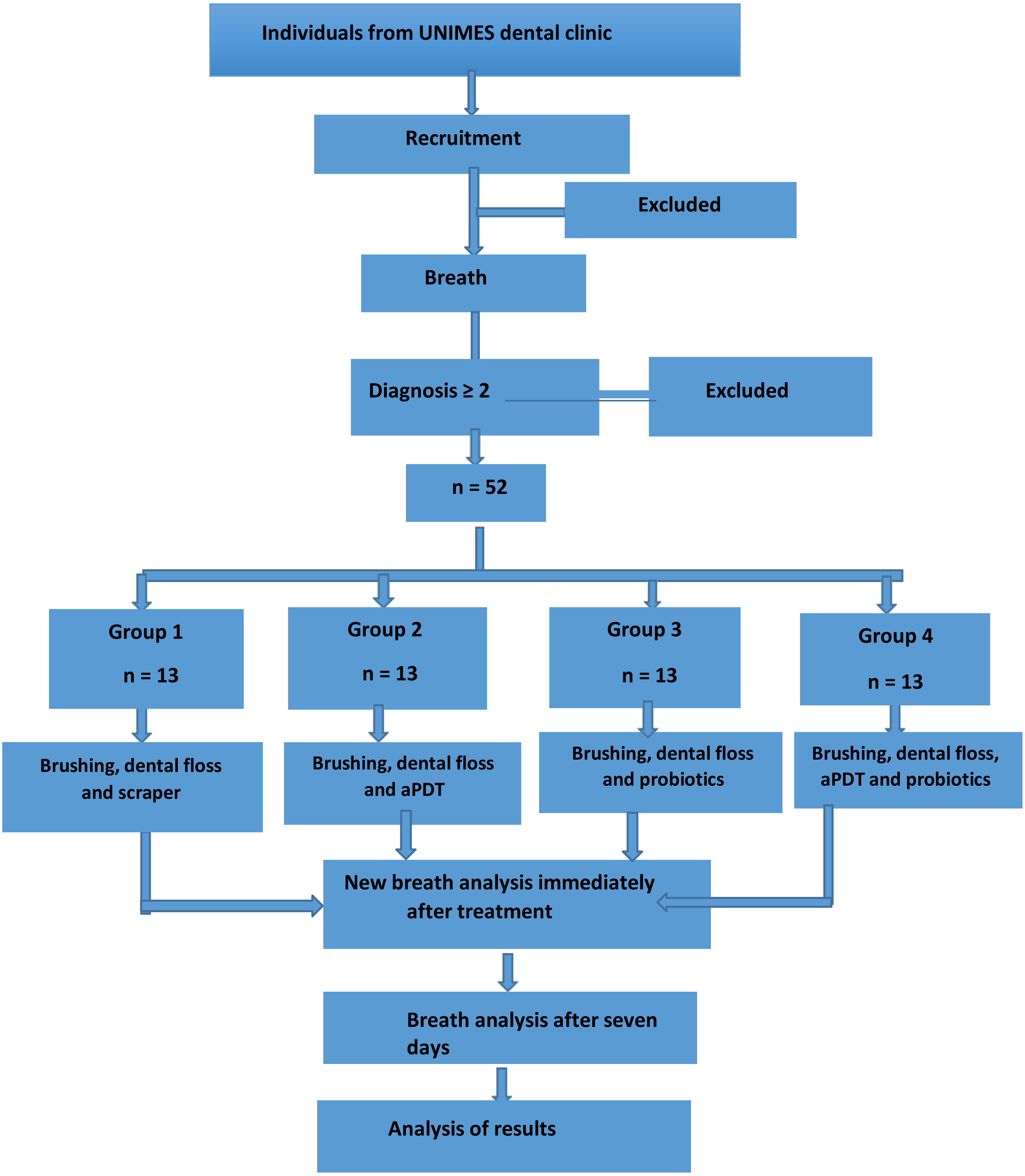

Figure 2: Flowchart of activities.

Coated tongue analysis

The quantity of tongue coating will be defined using the Coated Tongue Index proposed by Shimizu et al. [30]. The tongue is divided into nine parts, each of which receives a score: 0 – absence of coated tongue, 1 – thin tongue coating with visible papillae, 2 – thick tongue coating, papillae not visible.

Breath analysis

Air from the oral cavity will be collected following the manufacturer's orientations. The Breath-Alert device will be disinfected after each use. The device will be shaken four or five times prior to use to eliminate any residual odors. A "beep" is emitted upon opening the upper compartment of the device and a second "beep" is emitted when the volunteer blows into the front air input (passage of airflow). After a third "beep", the odor of the breath is measured and scored on a scale of 0 to 5 points. An "E" appears when an error has occurred and the procedure is repeated (Figure 3). A score of ≥ 2 points is considered indicative of halitosis.

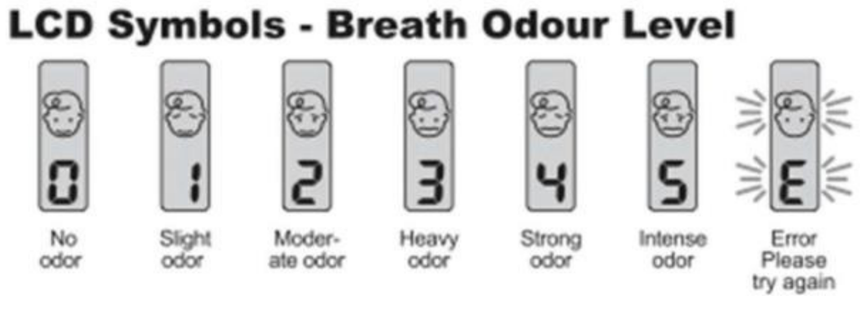

Figure 3: Breath odor analysis with breath meter.

To avoid the influence on the breath analysis results, the participants will be instructed to obey the following: in the 48 hours prior to the evaluation, avoid foods with garlic, onion, strong spices, the consumption of alcohol and the use of an oral antiseptic; on the day of the evaluation, abstain from all food in the two hours prior to the exam, abstain from coffee, mints, chewing gum, oral and personal hygiene products with perfume (aftershave, deodorant, perfume, creams and tonic) and brush only with water.

Microbiological analysis

Microbiological collections will be performed before and immediately after treatment. Samples of the tongue coating will be collected with a swab, soaked in reduced Amies medium. The samples will be taken to the laboratory for analysis in less than two hours after collection. The samples will be vortexed for approximately one minute. After homogenization, ten serial dilutions will be prepared in 180 µl of sterile phosphate buffered saline. Aliquots of 10^{-1}, 10^{-2}, 10^{-3}, 10^{-4} and 10^{-5} will be transferred to Petri dishes with sheep blood agar. As the main bacteria responsible for the production of VSCs are anaerobic, the Petri dishes will be incubated in anaerobiosis for 72 h at 37°C. Bacterial counts will be based on colony-forming units (CFUs) [4,22].

Microbiological analysis – real-time PCR

The samples of coated tongues will be collected with a sterile swab that will be placed in contact with the surface of the dorsum of the tongue with back-and-forth movements. The samples will be placed in tubes containing Tris–EDTA buffer (10 mM Tris – HCL, 0.1 mM EDTA, pH 7.5). The tubes will be duly labeled and stored at -80 °C until analysis. Maximum storage time will be one week. The samples will be frozen due to the impossibility of performing all analyses on a single day [3]. After thawing, the samples will be vortexed for one minute. The Master Pure DNA Extraction kit (Epicentre Technologies, Chicago, Illinois, USA) will be used for the extraction of

bacterial DNA. First, 100 μL of sample will be eluted in Tris-EDTA buffer. The samples will be centrifuged at 10,000 rpm and 4º C for 10 minutes. Next, the MIX will be prepared by diluting 300 μl of TCL + 2 μl of Prot K for each sample. The supernatant will be removed with a pipette, with a volume of 25 μl + the formed pellet remaining. Vortexing will be performed for 10 s to resuspend the pellet. A total of 302 μl of MIX will be added to each sample, followed by vortexing for 10 s and incubation at 65 ºC for 15 min with vortexing every five minutes. The samples will be placed in ice for 5 min. A total of 150 μl of MPC (protein precipitation reagent) will be added to the samples, followed by vortexing for 10 seconds. The samples will be centrifuged at 10,000 rpm and 4 °C for 10 minutes. The supernatant will be removed and placed in a new tube, to which 25 μl of MPC (protein precipitation) will be added. The tube will be centrifuged at 10,000 rpm and 4 °C for 10 minutes. A total of 500 μl of isopropanol will be added and inverted 40x, followed by centrifugation at 10,000 rpm and 4 °C for 10 minutes. The samples will be placed in ice without releasing the pellet. The isopropanol will be poured out and the rest will be removed with a 50-μl pipette. Washing will be performed with 200 μl of 70% ethanol carefully so as not do dislodge the pellet. Centrifugation will be performed at 10,000 rpm and 4 °C for 3 minutes. The residual ethanol will be removed and the sample will be left inverted to dry for 30 minutes. Elution will be performed in 40 μl of Tris-EDTA buffer, followed by freezing at -20 °C [32]. The purified DNA will be resuspended in Tris-EDTA buffer.

Levels of *P. gingivalis*, *T. forsythia* and *T. denticola* will be determined by quantitative PCR. The quantitative analysis will be performed by real-time PCR using a Step One Plus Real-Time PCR System thermocycler (Applied Biosystem, Foster City, CA, USA) and the products will be detected by fluorescence using the Quantimix Easy SYG Kit (Biotools, Madrid, Spain), following the manufacturer's protocol. For the reaction, 10 μl of SYBR Green, 0.5 μl of DNA mold and 200 mM of each primer (*P. gingivalis* CATAGATATCACGAGGAACTCCGA TT and AAACTGTTAGCAACTACCGATGTGG; *T. forsythia*

GGGTGAGTAACGCGTATGTAACCT and ACCCATCCGCAACCAATAAA; *T. denticola* CGTTCCTGGGCCTTGTACA and TAGCGACTTCAGGTACCCTCG;

and the universal primers for bacteria CCATGAAGTCGGAATCGCTAG and GCTTGACGGGCGGTGT) will be used in a total volume of 20 µl. Sterile Milli-Q water rather than DNA mold will be used as the negative control. Reactions for 16S rRNA will be performed with initial denaturation at 95 ºC for 2 minutes, followed by 36 cycles of 94 ºC for 30 seconds, 55 ºC for 1 minute and 72 ºC for 2 minutes and final extension at 72 ºC for 10 minutes. Fluorescence will be detected after each cycle and represented in a graph using the Step One Plus Real-Time PCR System (Applied Biosystem, Foster City, CA, USA). To ensure the specificity of the products detected by fluorescence and avoid the detection of primer dimers, detection will be performed one degree below the dissociation temperature of the amplicons. All samples will be analyzed in duplicate and each dilution of plasmids for the standard curve will be analyzed in triplicate. The purpose of the microbiological analysis will be to determine the effectiveness of photodynamic therapy for the treatment of halitosis, complementing the clinical assessment.

Antimicrobial photodynamic therapy (aPDT)

The Valo Cordless Ultradent® LED curing light will be used. This device has a coupled radiometer, spectrum of 440 to 480 nm and irradiance of 450 mW/cm. Only the volunteer being treated and operator of the device will be present at the time of aPDT, both of whom will be using protective eyewear. The active LED tip will be covered with disposable transparent plastic wrap (PVC) to avoid cross-contamination and for hygiene purposes and the operator will be duly garmented.

One session of aPDT will be performed with the annatto photosensitizer at a concentration of 20% (Fórmula e Ação®) in spray form to be applied in a sufficient quantity to cover the middle third and dorsum of the tongue (five sprays) with 2 minutes for incubation. The excess will be removed with an aspirator while maintaining the

surface moist with the photosensitizer without the use of water. Six points will be irradiated with a distance of 1 cm between points, considering the light scattering halo and effectiveness of aPDT. The device will be previously calibrated with a wavelength of 395 to 480 nm, 20 seconds per point and energy of 9.6 J. The light will be irradiated such that a halo will be 2 cm in diameter per point. Table 1 displays the parameters that will be used [31].

Table 1: LED parameters.

**Wavelength (nm)**	395-480
**Operating mode**	Continuous
**Mean radiant power (mW)**	480
**Polarization**	Random
**Aperture diameter (cm)**	0.9
**Irradiance at aperture (mW/cm^2)**	762
**Beam profile**	Top hat
**Irradiated area (cm^2)**	3.14
**Irradiance at target (mW/cm^2)**	153
**Exposure time (s)**	20

**Fluence (J/cm²)**	6.37
**Radiant energy (J)**	9.6
**Number of points irradiated**	6
**Total area irradiated (cm²)**	18.8
**Number of sessions**	1
**Total radiant energy (J)**	57.6

Tongue scraping

Tongue scraping will be performed by the same operator on all participants using posteroanterior movements of the scraper on the dorsum of the tongue, followed by cleaning of the scraper with gauze. This procedure will be performed ten times on each patient to standardize the mechanical removal of the tongue coating.

Treatment with probiotics

Pharmaceuticals (capsules or chewable gum) will be prepared at a compounding pharmacy. The products will contain strains of *Lactobacillus salivarius* WB21 (6.7 x 10^8 CFUs) and xylitol (280 mg). Each patient should take the pharmaceutical three times per day after meals for seven days.

Brushing with toothpaste containing amine fluoride

All 52 participants will be instructed to brush with a toothpaste containing amine fluoride (Elmex®) and use dental floss three times per day after meals for 10 days.

Organization and statistical treatment of data

Data from the portable Breath-Alert™ device will be analyzed for normality using the Shapiro–Wilk test. Parametric data will be submitted to analysis of variance (ANOVA) followed by Tukey's test when necessary and the t-test will be used for paired data to analyze the results of treatment in the two periods of the study. Nonparametric data will be compared using the Kruskal–Wallis test followed by the Student–Newman–Keuls

test when necessary and the Wilcoxon text will be used for the comparison of each treatment in the two periods of the study.

3. Expected results

Through this study, we will be able to determine whether there are differences among the proposed treatments. We will also determine whether a reduction in halitosis occurs after the use of photodynamic therapy involving the use of annatto as the photosensitizer and blue LED as the light source and after treatment with probiotics.

REFERENCES

1. BAWAZIR O. A. (2021). Risk Factors, Diagnosis, and Management of Halitosis in Children: A Comprehensive Review. The journal of contemporary dental practice, 22(8), 959–963.
2. PORTER, S. R.; SCULLY, C. Oral malodour (halitosis). Bmj, v. 333, n. 7569, p. 632-635, 2006.
3. BICAK, Damla Aksit. A current approach to halitosis and oral malodor-A mini review. The open dentistry journal, v. 12, p. 322, 2018.
4. ELIAS, Marina Sá; FERRIANI, Maria das Graças Carvalho. Aspectos históricos e sociais da halitose. Revista Latino-Americana de Enfermagem, v. 14, n. 5, 2006.
5. Guedes, C. C., Bussadori, S. K., Garcia, A., Motta, L. J., Gomes, A. O., Weber, R., & Amancio, O. (2020). Accuracy of a portable breath meter test for the detection of halitosis in children and adolescents. Clinics (Sao Paulo, Brazil), 75, e1764. https://doi.org/10.6061/clinics/2020/e1764
6. AlMadhi, N.A.; Sulimany, A.M.; Alzoman, H.A.; Bawazir, O.A. Halitosis in Children Undergoing Full Mouth Rehabilitation under General Anesthesia. Children 2021, 8, 149. https://doi.org/10.3390/children8020149.
7. Małgorzata Sikorska-Żuk and Marek Bochnia Halitosis in children with adenoid

hypertrophy, 2018 J. Breath Res. 12 026011.

8. Alqutami, J., Elger, W., Grafe, N., Hiemisch, A., Kiess, W., & Hirsch, C. (2019). Dental health, halitosis and mouth breathing in 10-to-15 year old children: A potential connection. European journal of paediatric dentistry, 20(4), 274–279. https://doi.org/10.23804/ejpd.2019.20.04.03.
9. Bawazir O. A. (2021). Risk Factors, Diagnosis, and Management of Halitosis in Children: A Comprehensive Review. The journal of contemporary dental practice, 22(8), 959–963.
10. Motta LJ, Bachiega JC, Guedes CC, Laranja LT, Bussadori SK. Association between halitosis and mouth breathing in children. Clinics (Sao Paulo). 2011;66(6):939-942. doi:10.1590/s1807-59322011000600003.
11. İnönü-Sakallı N, Sakallı C, Tosun Ö, Akşit-Bıçak D. Comparative Evaluation of the Effects of Adenotonsillar Hypertrophy on Oral Health in Children. Biomed Res Int. 2021;2021:5550267. Published 2021 Apr 2. doi:10.1155/2021/5550267
12. KARA, C; TEZEL, A; ORBAK, R. Effect of oral hygiene instruction and scaling on oral malodour in a population of Turkish children with gingival inflammation. Int J Paediatr Dent, v. 16, n. 6, p. 399–404, nov. 2006.
13. Eli I, Koriat H, Baht R Rosenberg M. Self-perception of breath odor: role of body image and psychopathologic traits. Percept Mot Skills. 2000;91 (3 pt 2):1193-201, doi: 10.2466/PMS.91.7.1193-1201. 9. Yaegaki K, Coil JM. Examination, classification, and treatment of halitosis; clinical perspectives. J Can Dent Assoc. 2000;66:257-61. 10.
14. Riggio MP, Lennon A, Rolph HJ, Hodge PJ, Donaldson A, Maxwell AJ, et al. Molecular identification of bacteria on the tongue dorsum of subjects with and without halitosis. Oral Dis. 2008;14:251-8, doi: 10.1111/ j.1601-0825.2007.01371.x.
15. TOLENTINO, E. D. S.; CHINELLATO, L. E. M.; TARZIA, O. Saliva and tongue coating pH before and after use of mouthwashes and relationship with parameters of halitosis. J Appl Oral Sci, v. 19, n. 2, p. 90–4, abr. 2011.

16. DA CIARCIA, Ana Carolina Costa et al. Action of antimicrobial photodynamic therapy with red leds in microorganisms related to halitose. Medicine, v. 98, n. 1, 2019.
17. YOO, Jun-Il et al. The Effect of Probiotics on Halitosis: a Systematic Review and Meta-analysis. Probiotics and antimicrobial proteins, v. 11, n. 1, p. 150-157, 2019.
18. CALIL, CM.; MARCONDES, FK. Influence of anxiety on the production of oral volatile sulfur compounds. Life Science, v. 79, n. 7, p. 660–4, 10 jul. 2006.
19. SPRINGFIELD, J. et al. Spontaneous fluctuations in the concentrations of oral sulfurcontaining gases. J Dental Res, v. 80, n. 5, p. 1441–1444, 2001.
20. TANGERMAN, A; WINKEL, E. G. The portable gas chromatograph OralchromaTM: a method of choice to detect oral and extra-oral halitosis. J Breath Res, v. 2, n. 1, mar. 2008.
21. KARA, C. et al. Effect of Nd: YAG laser irradiation on the treatment of oral malodour associated with chronic periodontitis. Int Dent J, v. 58, p. 151–158, 2008.
22. COSTA DA MOTA, Ana Carolina et al. Effect of photodynamic therapy for the treatment of halitosis in adolescents–a controlled, microbiological, clinical trial. Journal of biophotonics, v. 9, n. 11-12, p. 1337-1343, 2016.
23. López-Valverde N, López-Valverde A, Macedo de Sousa B, Rodríguez C, Suárez A and Aragoneses JM (2022) Role of Probiotics in Halitosis of Oral Origin: A Systematic Review and Meta-Analysis of Random
24. Motta, P. B., Motta, L. J., Campos, T. M., Gonçalves, M., Santos, E. M., Martimbianco, A., de Andrade, D., Mesquita-Ferrari, R. A., Fernandes, K., Horliana, A., & Bussadori, S. K. (2022). Effect of Photodynamic Therapy on Halitosis: A Systematic Review of Randomized Controlled Trials. Sensors (Basel, Switzerland), 22(2), 469. https://doi.org/10.3390/s22020469
25. HOPE, Chris K.; WILSON, M. Induction of lethal photosensitization in biofilms using a confocal scanning laser as the excitation source. Journal of Antimicrobial Chemotherapy, v. 57, n. 6, p. 1227-1230, 2006.

26. SALMINEN, Seppo et al. Demonstration of safety of probiotics—a review. International journal of food microbiology, v. 44, n. 1-2, p. 93-106, 1998.
27. SUZUKI, Nao et al. Induction and Inhibition of Oral Malodor. Molecular Oral Microbiology, 2019.
28. KELLESARIAN, Sergio Varela et al. Effect of antimicrobial photodynamic therapy and laser alone as adjunct to mechanical debridement in the management of halitosis: A systematic review. Quintessence International, v. 48, n. 7, 2017.
29. Vilar Daniela de Araújo, Vilar Marina Suênia de Araujo, Moura Túlio Flávio Accioly de Lima e, Raffin Fernanda Nervo, Oliveira Márcia Rosa de, Franco Camilo Flamarion de Oliveira, de Athayde-Filho Petrônio Filgueiras, Diniz Margareth de Fátima Formiga Melo, Barbosa-Filho José Maria. Traditional Uses, Chemical Constituents, and Biological Activities ofBixa orellanaL.: A Review. The Scientific World Journal. 2014;2014:1–11. doi: 10.1155/2014/857292.
30. Shimizu T, Ueda T and Sakurai K 2007 New method for evaluation of tongue-coating status J. Oral Rehabil. 34 442–.
31. GONÇALVES, Marcela Leticia Leal et al. Photodynamic therapy with Bixa orellana extract and LED for the reduction of halitosis: study protocol for a randomized, microbiological and clinical trial. Trials, v. 19, n. 1, p. 590, 2018.
