# Supplementary material for: Assessment of photodynamic therapy with annatto and led for the treatment of halitosis in mouth-breathing children: Randomized controlled clinical trial
Source: PLoS One. 2024 Sep 3;19(9):e0307957. doi: 10.1371/journal.pone.0307957 (PMC11371243; doi:10.1371/journal.pone.0307957)
Supplement: S6 File — (PDF) [file pone.0307957.s007.pdf]

TERMO DE CONSENTIMENTO LIVRE E ESCLARECIDO – (TCLE)

TERMO DE CONSENTIMENTO LIVRE E ESCLARECIDO

ESTUDO COMPARATIVO ENTRE A TERAPIA FOTODINÂMICA E USO DE PROBIÓTICOS NA REDUÇÃO DA HALITOSE EM CRIANÇAS RESPIRADORAS ORAIS: ENSAIO CLÍNICO CONTROLADO E RANDOMIZADO

Número do CAAE:

Você está sendo chamado a participar como voluntário de uma pesquisa cujo estudo é **ESTUDO COMPARATIVO ENTRE A TERAPIA FOTODINÂMICA E USO DE PROBIÓTICOS NA REDUÇÃO DA HALITOSE EM CRIANÇAS RESPIRADORAS ORAIS: ENSAIO CLÍNICO CONTROLADO E RANDOMIZADO.** Este documento, chamado Termo de Consentimento Livre e Esclarecido, visa assegurar seus direitos como participante e é elaborado em duas vias, uma que deve ficar com você e outra com o pesquisador.

Por favor, leia com atenção e calma. Se perguntas antes ou mesmo depois de assiná-lo, você poderá declarar-las com o pesquisador. Não haverá nenhum tipo de penalização ou prejuízo se você não aceitar sua autorização em qualquer um.

Justificativa e objetivos:

Justificativa é um tema que ainda está atento aos resultados: O tratamento da saúde tem uma tomada de decisões dos profissionais e já à precisa PDT usando LEDs azuis para tratar a saúde em seu cotidiano que a maioria dos dentistas possui essa fonte de luz em seus já possuidores e o portátil é custo para aquisição. Além disso, a utilização do extrato urucum como fotossensibilizador é inovador. Uma vez que se trata de uma fonte de luz e um sensibilizador acessível, espera-se que este tratamento seja considerado clinicamente com rapidez e facilidade. Espera-se que o uso de probióticos continue a aumentar o uso do aPDT em crianças e o uso de respiradoras orais.

Objetivos: O objetivo do presente estudo é verificar se o tratamento com aPDT , usando extrato de urucum como fotossensibilizador e LED azul como fonte de luz, é eficaz na redução em crianças respiradoras orais.

Procedimentos:

A pesquisa será realizada com pacientes de ambos os sexos matriculados regularmente na Clínica de Odontologia da Universidade Metropolitana de Santos (UNIMES).

O tipo de tratamento será determinado aleatoriamente para cada dente, através da realização de um sorteio antes da intervenção.

Grupo 1: tratamento com escovação, fio dental e raspador de língua;
Grupo 2: escovação, fio dental e aPDT aplicado na região de dorso médio da língua;
Grupo 3: escovação fio dental e probióticos;
Grupo 4: escovação, fio dental, aPDT e probióticos.

RUBRICA DO PARTICIPANTE DA PESQUISA

RUBRICA DO PESQUISADOR

Desconfortos e riscos:

Os riscos relacionados aos procedimentos estão ligados a possíveis constrangimentos ao responder perguntas, desconfortos durante os tratamentos e sintomatologia dolorosa. Para minimizar os riscos, os problemas serão realizados em questões reservadas, realizarão os procedimentos com a maior rapidez e serão realizados via telefone para perguntas, saber ou para questões reservadas. Nos procedimentos com laser, os riscos a visão serão minimizados com a utilização de óculos de proteção adequada.

Benefícios:

Os voluntários e seus responsáveis participam das atividades de educação em saúde bucal com aconselhamento de alimentação e higiene. Os voluntários terão a bocada e se houver necessidade de serem examinados para tratamento.

Acompanhamento e assistência:

Um momento, antes, durante ou até o termo da pesquisa, nós colocamos em dúvida a provisão para qualquer esclarecimento de qualquer.

Sigilo e privacidade:

Você tem a garantia de que sua identidade será mantida em sigilo. Os dados da natureza e os eventos foram divulgados, que podem ser apresentados em sua identidade/ou divulgados, sem revelar a identidade dos eventos.

RUBRICA DO PARTICIPANTE DA PESQUISA

RUBRICA DO PESQUISADOR

Ressarcimento e Indenização:
Caso esta causa, comprovadamente, qualquer pesquisa ou dano procure o pesquisador responsável a fim de ressarcimento ou possível indenização.

Contato:
Em caso de dúvidas sobre a pesquisa, se precisar consultar esse registro de consentimento ou quaisquer outras questões, você poderá entrar em com os pesquisadores:

Nome do pesquisador responsável:
Endereço: Ana Paula Taboada Sobral
E-mail: anapaula@taboada.com.br

Nome do pesquisador
Endereço:
Telefone:
E-mail:

Caso de denúncias ou às reclamações sobre sua participação e questões sobre sua participação, você poderá entrar em contato com o Comitê de Ética em Pesquisa da Universidade Metropolitana de Santos (das 08h30 às 11h30 e das 13h00) na Avenida Conselheiro Nébias , 536 - 2.º andar. Santos-SP. E-mail: cpq@unimes.br

Consentimento Livre e Esclarecido:
ter recebido esclarecimentos sobre a natureza da pesquisa, seus objetivos, procedimentos, benefícios previstos, possíveis riscos e o problema após este estudo poder participar, aceito:

Nome do(a) participante: ____

_____________________________________ Dados: ____/_____/______.
(Assinatura do participante ou nome e assinatura do seu RESPONSÁVEL LEGAL)

Responsabilidade do Pesquisador:
Asseguro ter sido explicado e fornecido via documento ao participante. Informa que o estudo foi aprovado pelo CEP perante o qual o projeto foi apresentado. Comprometa-se a usar o material e os dados obtidos nesta pesquisa exclusivamente para fins de previsão neste documento ou conforme o consentimento dado pelo participante.

___________________________________. Dados: ____/_____/______.
(Assinatura do pesquisado

RUBRICA DO PARTICIPANTE DA PESQUISA

RUBRICA DO PESQUISADOR
