# Supplementary material for: Assessment of photodynamic therapy with annatto and led for the treatment of halitosis in mouth-breathing children: Randomized controlled clinical trial
Source: PLoS One. 2024 Sep 3;19(9):e0307957. doi: 10.1371/journal.pone.0307957 (PMC11371243; doi:10.1371/journal.pone.0307957)
Supplement: S7 File — (PDF) [file pone.0307957.s008.pdf]

FREE AND CLARIFIED CONSENT TERM

COMPARATIVE STUDY BETWEEN PHOTODYNAMIC THERAPY AND THE USE OF PROBIOTICS IN THE REDUCTION OF HALITOSIS IN ORAL BREATHING CHILDREN: RANDOMIZED CONTROLLED CLINICAL TRIAL
CAAE number:

You are being invited to participate as a volunteer in a research whose title is **COMPARATIVE STUDY BETWEEN PHOTODYNAMIC THERAPY AND THE USE OF PROBIOTICS IN THE REDUCTION OF HALITOSIS IN ORAL BREATHING CHILDREN: RANDOMIZED CONTROLLED CLINICAL TRIAL.** This document, called the Free and Informed Consent Term, aims to ensure your rights as a participant and is prepared in two copies, one that must be with you and the other with the researcher.

Please read carefully and calmly. If there are questions before or even after signing it, you can clarify them with the researcher. There will be no penalty or detriment if you do not accept to participate or withdraw your authorization at any time.

Justification and objectives:
Rationale: The treatment of halitosis is a topic that still needs attention and the results of this study can support the decision-making of healthcare professionals regarding the use of probiotics and aPDT using blue LEDs to treat halitosis in their daily lives, since that most dentists already have this light source in their offices and the portable meter is inexpensive to purchase. In addition, the use of annatto extract as a photosensitizer is innovative. As it is an affordable light source and photosensitizer, it is expected that this treatment will be clinically reproduced with effectiveness and ease. The use of probiotics and the use of aPDT are expected to be effective in decreasing halitosis in mouth breathing children.

Objectives: The objective of the present study is to verify if the treatment with aPDT , using annatto extract as a photosensitizer and blue LED as a light source, is effective in reducing mouth breathing children.

Procedures:

The research will be carried out with patients of both sexes regularly enrolled in the Dental Clinic of the Metropolitan University of Santos (UNIMES).

The type of treatment will be randomly determined for each tooth, through a lottery before the intervention.

Group 1: treatment with brushing, dental floss and tongue scraper;
Group 2: brushing, dental floss and aPDT applied to the dorsum and middle third of the tongue;
Group 3: brushing, flossing and probiotics;
Group 4: brushing, dental floss, aPDT and probiotics.

RUBRICA DO PARTICIPANTE DA PESQUISA

RUBRICA DO PESQUISADOR

Discomforts and risks:

The risks related to the procedures are linked to possible embarrassment when answering questions, discomfort during treatments and possible painful symptoms. To minimize the risks, the researchers will ask questions in reserved environments, will carry out the procedures as quickly as possible and will be available via telephone to answer questions or for emergencies. In laser procedures, the risks to vision will be minimized with the use of suitable protective eyewear.

Benefits:

Volunteers and their guardians will participate in oral health education activities with food and hygiene advice. Volunteers will have their mouths examined and, if necessary, will be referred for dental treatment.

Follow-up and assistance:

At any time, before, during or until the end of the research, we are available to clarify any doubts about the research.

Secrecy and privacy:

You are assured that your identity will be kept confidential. The data collected will be used exclusively for research purposes, which may be presented at scientific and/or published events, without revealing the identity of the participants.

RUBRICA DO PARTICIPANTE DA PESQUISA

RUBRICA DO PESQUISADOR

Reimbursement and Indemnity:
If this research demonstrably causes any cost or damage, look for the responsible researcher for reimbursement or possible compensation.

Contact:
If you have questions about the research, if you need to consult this consent record or any other questions, you can contact the researchers:

Name of the responsible researcher:
Address: Ana Paula Taboada Sobral
Email: anapaula@taboada.com.br

Research student name
Address:
Telephone:
Email:

In case of complaints or complaints about your participation and about ethical issues of the study, you can contact the secretary of the Research Ethics Committee of the Metropolitan University of Santos (from 8:30 am to 11:30 am and from 1:00 pm to 5:00 pm) at Avenida Conselheiro Nébias , 536 - 2nd floor. Santos - SP. Email: cpq@unimes.br

Free and Informed Consent:
After having received clarification on the nature of the research, its objectives, procedures, expected benefits, potential risks and the inconvenience that this study may cause, I accept to participate:

Name of participant: _______________________________________

_______________________________________ Date: ____/_____/______.
(Signature of the participant or name and signature of their LEGAL RESPONSIBLE)

Researcher Responsibilities:
I assure you that I have explained and provided a copy of this document to the participant. I inform you that the study was approved by the CEP before which the project was presented. I undertake to use the material and data obtained in this research exclusively for the purposes set out in this document or in accordance with the consent given by the participant.

_____________________________________ Date: ____/_____/______.
(Signature of the researched

RUBRICA DO PARTICIPANTE DA PESQUISA

RUBRICA DO PESQUISADOR
