# Supplementary material for: Assessment of photodynamic therapy with annatto and led for the treatment of halitosis in mouth-breathing children: Randomized controlled clinical trial
Source: PLoS One. 2024 Sep 3;19(9):e0307957. doi: 10.1371/journal.pone.0307957 (PMC11371243; doi:10.1371/journal.pone.0307957)
Supplement: S8 File — (PDF) [file pone.0307957.s009.pdf]

ClinicalTrials.gov Protocol Registration and Results System (PRS) Receipt
Release Date: March 28, 2024

ClinicalTrials.gov ID: NCT05590897

Study Identification

Unique Protocol ID: MouthBreathing

Brief Title: Assessment of Photodynamic Therapy With Annatto and Led for the Treatment of Halitosis in Mouth-Breathing Children

Official Title: Assessment of Photodynamic Therapy With Annatto and Led for the Treatment of Halitosis in Mouth-Breathing Children: Randomized Controlled Clinical Trial

Secondary IDs:

Study Status

Record Verification: March 2024

Overall Status: Completed

Study Start: February 1, 2023 [Actual]

Primary Completion: October 1, 2023 [Actual]

Study Completion: December 20, 2023 [Actual]

Sponsor/Collaborators

Sponsor: University of Nove de Julho

Responsible Party: Principal Investigator
Investigator: Sandra Kalil Bussadori [sbussadori]
Official Title: Principal Investigator
Affiliation: University of Nove de Julho

Collaborators:

Oversight

U.S. FDA-regulated Drug: No

U.S. FDA-regulated Device: No

U.S. FDA IND/IDE: No

Human Subjects Review: Board Status: Approved
Approval Number: 64510922.6.0000.5509
Board Name: Universidade Metropolitana de Santos Ethics Committee
Board Affiliation: Universidade Metropolitana de Santos
Phone: +55 13 32283400
Email: fernanda.agnelli@unimes.br
Address:

Gen. Francisco Glycerio Avenue, 8 - Encruzilhada, Santos - SP, Brazil, ZIP -11045-002

Data Monitoring:

Study Description

Brief Summary: Objective: To assess the effectiveness of antimicrobial photodynamic therapy (aPDT) employing an annatto-based (20%) dye combined with blue LED for the treatment of halitosis in mouth-breathing children. Methods: Fifty-two children six to twelve years of age with diagnoses of mouth breathing and halitosis (score of ≥ 3 on Breath Alert® portable breath meter) were randomly allocated to two groups (n = 26). Group 1: brushing, dental floss and aPDT applied to middle third of the dorsum of the tongue. Group 2: brushing, dental floss and tongue scraper. Breath meter results before, immediately after treatment as well as seven and 30 days after treatment were compared. The normality of the data will be determined using the Shapiro–Wilk test. Parametric data will be submitted to analysis of variance and nonparametric data will be compared using the Kruskal–Wallis test. The results of each treatment in the different periods of the study will be compared using the Wilcoxon test.

Detailed Description:

Conditions

Conditions: Halitosis

Keywords:

Study Design

Study Type: Interventional

Primary Purpose: Treatment

Study Phase: Phase 1/Phase 2

Interventional Study Model: Parallel Assignment

Number of Arms: 2

Masking: Single (Outcomes Assessor)
The Breath Alert, as the device that will be used to determine the presence of halitosis, is blinded to groups/participants.

Allocation: Randomized

Enrollment: 52 [Actual]

Arms and Interventions

Arms	Assigned Interventions
Experimental: Antimicrobial Photodynamic Therapy Group<br>Participants in this group will receive treatment with tooth brushing, dental flossing and antimicrobial photodynamic therapy in the tongue.	Procedure/Surgery: Teeth Brushing<br>All 52 participants were instructed through a lecture on how to perform toothbrushing with a fluoridated toothpaste (Colgate T®) and dental flossing three times per day after meals for 30 days. The Bass technique was taught, by which the bristles should be positioned at an approximate angle of 45º to the gingival pocket on both the free and proximal faces,

Arms	Assigned Interventions
	with the performance of short, slightly circular vibrating movements.<br>Procedure/Surgery: Dental Flossing<br>All 52 participants were instructed to floss, 3 times a day after meals for 30 days.<br>Radiation: Antimicrobial Photodynamic Therapy (aPDT)<br>One session of aPDT was performed. The annatto photosensitizer was mixed at a concentration of 20% (Fórmula e Ação®, Brazil) in spray form, applied with a sufficient quantity to coat the middle third of the dorsum of the tongue (five sprays) and left for two minutes for incubation. The excess was removed with an aspirator. |The surface of the tongue remained moist with the photosensitizer itself without the use of water. Six points were irradiated with a distance of 1 cm between points, considering the spread of the light and effectiveness of aPDT. The device was previously calibrated at a wavelength of 395-480 nm. Light was irradiated to ensure a beam area of 2 cm in diameter per point. Energy was 9.6 J and exposure time was 20 seconds per point.
Experimental: Tongue Scraping Group<br>Participants in this group will receive treatment with tooth brushing, dental flossing and tongue scraping.	Procedure/Surgery: Teeth Brushing<br>All 52 participants were instructed through a lecture on how to perform toothbrushing with a fluoridated toothpaste (Colgate T®) and dental flossing three times per day after meals for 30 days. The Bass technique was taught, by which the bristles should be positioned at an approximate angle of 45º to the gingival pocket on both the free and proximal faces, with the performance of short, slightly circular vibrating movements.<br>Procedure/Surgery: Dental Flossing<br>All 52 participants were instructed to floss, 3 times a day after meals for 30 days.<br>Procedure/Surgery: Tongue Scraping<br>Tongue scraping was performed by the same operator for all participants. Posteroanterior movements were performed with the scraper on the dorsum of the tongue, followed by the cleaning of the scraper with gauze. The procedure was performed ten times on each participant to standardize mechanical removal.

Outcome Measures

Primary Outcome Measure:

1. Changes in Halimetry Results
   The Breath Alert® device was used following the manufacturer’s instructions and disinfected after each use. The device was shaken four or five times prior to each use to eliminate any residual odors. A “beep” was emitted upon opening the upper compartment of the device and a second “beep” was emitted when the participant blew into the frontal air input (air flow passage). After a third “beep”, breath odor was measured and scored on a scale of 0 to 8 points. If the letter ‘’C’’ appeared, indicating an error, the procedure was repeated. A score ≥ 2 points was considered indicative of halitosis.

   [Time Frame: Baseline, immediately after treatment, after 7 days and after 30 days.]

Eligibility

Minimum Age: 6 Years

Maximum Age: 12 Years

Sex: All

Gender Based:

Accepts Healthy Volunteers: No

Criteria: Inclusion Criteria:

- Diagnosis of mouth breathing (performed using the calibrated Glatzel mirror test, water retention test and a questionnaire);
- Halitosis presenting a score equal or greater than 2, in the Breath Alert analysis.

Exclusion Criteria:

- Nasal breathers;
- Dentofacial anomalies (such as cleft lip, cleft palate and nasopalatine);
- Undergoing orthodontic and/or orthopedic treatment;
- Undergoing cancer treatment;
- Systemic alterations (gastrointestinal, renal, hepatic);
- Under antibiotic treatment for up to 1 month before the research;
- Fissured or grooved tongue.

Contacts/Locations

Central Contact Person:

Central Contact Backup:

Study Officials:

Locations: **Brazil**
Universidade Metropolitana de Santos - UNIMES
Santos, São Paulo, Brazil, 11045-002
Contact: Sandra Kalil sandra.skb@gmail.com

IPDSharing

Plan to Share IPD:

References

Citations:

Links:

Available IPD/Information:
